# Exploration of Cyberethics in Health Professions Education: A Scoping Review

**DOI:** 10.3390/ijerph20227048

**Published:** 2023-11-10

**Authors:** Jennie C. De Gagne, Eunji Cho, Paige S. Randall, Hyeyoung Hwang, Emily Wang, Leeho Yoo, Sandy Yamane, Leila S. Ledbetter, Dukyoo Jung

**Affiliations:** 1 School of Nursing, Duke University, Durham, NC 27710, USA; paige.synesael@duke.edu; 2 Connell School of Nursing, Boston College, Chestnut Hill, MA 02467, USA; eunji.cho@bc.edu; 3 Adventist HealthCare Shady Grove Medical Center, Rockville, MD 20850, USA; hwanghy.grace@gmail.com; 4 Duke University Health System, Durham, NC 27710, USA; emily.wang957@duke.edu; 5 College of Nursing, Ewha Womans University, Seoul 03760, Republic of Korea; haha_riho@naver.com (L.Y.); dyjung@ewha.ac.kr (D.J.); 6 School of Nursing, University of North Carolina at Greensboro, Greensboro, NC 27402, USA; ssyamane@uncg.edu; 7 Medical Center Library, Duke University, Durham, NC 27708, USA; leila.ledbetter@duke.edu

**Keywords:** cyberethics, cybercivility, health professions education, scoping review, pedagogy, theoretical frameworks, digital professionalism, e-professionalism, cultural sensitivity, emerging technologies

## Abstract

As digital technologies rapidly integrate into Health Professions Education (HPE), understanding cyberethics is increasingly crucial. This scoping review explores the pedagogy of cyberethics in HPE, highlighting a significant gap in explicit definitions and conceptualizations. Additionally, the absence of specific theoretical frameworks in most documents raises concerns about research progression. Only four articles introduce educational interventions in cyberethics, indicating a promising avenue for future research. While comprehensive search methods are employed, limitations, including language biases, exist. Future investigations should broaden the discourse to encompass ethical implications of emerging technologies within HPE. Cultivating comprehensive, culturally sensitive, and inclusive guidelines is vital for ethical digital practices in the health care community.

## 1. Introduction

Health Professions Education (HPE) has been transformed in recent years by the integration of digital technologies, internet applications, and social media platforms [1,2,3]. Although these advancements have propelled the field forward by offering new opportunities, they have also introduced some complex ethical and professional dilemmas related to educational curricula and pedagogical practices [4,5,6]. One such challenge is the escalating phenomenon of cyberincivility, or online conduct that is problematic or disrespectful [7,8,9]. The shift to online communication and learning necessitated by the COVID-19 pandemic accentuated this issue, increasing its significance [5]. Incidents such as the termination of nurses for posting an inappropriate video online [10] highlight the importance of enhancing educational efforts on cybercivility and fostering a deeper understanding of cyberethics in health care settings.

Emphasis has been consistently placed on the teaching of cybercivility, which promotes respectful online interactions [9]; however, addressing cyberincivility requires a dual approach that couples the promotion of cybercivility with a grounding in cyberethics—the applied ethics examining the ethical, legal, and social implications of cybertechnology [11]. The relationship between cybercivility and cyberethics reflects the broad interplay between civility and ethics [12,13]. Civility acts as a foundation for ethics, enabling society to reach its full potential; ethics addresses broader moral outcomes [13]. Consequently, cybercivility can be understood as a subset of cyberethics. Although both emphasize respectful online conduct, cybercivility primarily fosters positive interactions, whereas cyberethics facilitates responsible and ethical usage of technology [14]. This study aims to delve into cyberethics, concentrating on normative attributes, to enrich ethical competence in HPE.

The prefix “cyber” commonly denotes objects or ideas related to computers and the internet [15,16]. As cyberethics is closely linked to the development of internet technology, any definition of cyberethics involves online terminology [11,16]. Although the terms cyberethics and internet ethics have often been used interchangeably in the literature, internet ethics is a unique branch of computer ethics which deals specifically with behaviors performed on the internet [17,18]. Cyberethics encompasses a wide array of topics, including privacy, academic integrity, netiquette, the dissemination of false or inappropriate information, cyberbullying, online gambling, gaming, and internet addiction [15,19]. In this study, the term cyberethics (rather than internet ethics or computer ethics) is used to address the wide range of topics discussed in the literature.

The central objective of cyberethics education is to shape ethical principles that can effectively guide human behavior, thereby contributing to the establishment of a sustainable and inclusive global information society [19]. Notably, health professions students have exhibited a keen interest in digital professionalism and guidelines for safe online conduct [20]. However, there exists a noticeable gap in information regarding pedagogical strategies for integrating cyberethics into HPE [7,8,9]. Existing literature calls for focused efforts to provide a comprehensive model for the curricular development of cybercivility pedagogy [7,9] and to clarify policies and procedures to guide online behaviors and interactions among and between HPE faculty and students [7,8,9]. The exploration of cyberethics within HPE goes beyond academic pursuits—it has tangible real-world implications. As health care professionals increasingly turn to digital platforms for education, collaboration, and patient care, understanding and adhering to cyberethics principles become paramount.

This scoping review delves into the needs, scope, and philosophical underpinnings of cyberethics education, aiming to provide a comprehensive exploration of the existing literature on cyberethics in HPE. Given its relevance, this review seeks to map key concepts, identify gaps in knowledge, and elucidate the current state of cyberethics education in HPE. Specifically, we address the following research questions: (1) How has cyberethics been defined or conceptualized in the literature? (2) What tools (e.g., theories, models, conceptual or theoretical frameworks) have been identified in the literature to guide cyberethics education? and (3) If curricular interventions were employed, what methods of cyberethics education were described? With these questions as our foundation, the subsequent sections describe our methodology, explaining our approach in exploring these topics and charting the landscape of cyberethics literature within HPE.

## 2. Materials and Methods

The 5-step scoping review framework applied in this study was originally developed by Arksey and O’Malley [21] and Levac et al. [22] and updated by JBI guidelines for Scoping Reviews [23]. This framework consists of the following steps: (1) identify the research question and clarify the purpose of the scoping review; (2) conduct a comprehensive search for relevant studies; (3) iteratively select studies through searching, refining, and reviewing; (4) extract and chart the collected data; and (5) summarize and report the results [22]. To ensure systematic reporting, the Preferred Reporting Items for Systematic Review and Meta-Analyses Extension for Scoping Reviews (PRISMA-ScR) guidelines [24] were followed. An a priori protocol was developed and registered [25].

### 2.1. Stage 1: Identification of the Research Question

Our overarching research question for this scoping review was formulated using the PCC (Population/Problem, Concept, Context) framework proposed by Peters et al. [23]. The specific question addressed was “What is known from the exiting literature on cyberethics education for health professions students in learning environments?” The target population in question comprised health professions students, and the concept of interest was cyberethics education. The context of the study encompassed academic institutions worldwide, including universities and colleges that provide cyberethics education.

### 2.2. Stage 2: Identifying Relevant Studies

#### Search Strategy and Information Sources

To identify existing evidence synthesis on cyberethics education for health professions students, a preliminary search was conducted on PROSPERO and PubMed. These databases were chosen based on their reputability and comprehensiveness in hosting registered systematic reviews. However, no relevant results were found, indicating a potential gap in the literature. To ensure a thorough and comprehensive search strategy, professional medical librarians were consulted. Their expertise greatly aided in crafting a tailored search strategy, focusing on the nuances of English and Korean literature within the field. The following databases were searched for English literature: (1) Medline via PubMed, (2) Cumulative Index to Nursing and Allied Health Literature (CINAHL) via EBSCOhost, (3) PsycInfo via EBSCOhost, (4) Sociology Source Ultimate via EBSCOhost, and (5) Education Full Text via EBSCOhost. For Korean literature, the following databases were searched: (1) RISS of the Korea Education and Research information Service, (2) Korean studies Information Service System (KISS), (3) DBpia (i.e., multidisciplinary full-text database of journal articles published by major Korean research institutions), and (4) ScienceON via OOO University library. In addition, grey literature searches were conducted using ProQuest Dissertations and Theses Global database (English) and RISS (Korean). Grey literature documents were searched on relevant English and Korean websites as well as websites of professional organizations. Such websites often house guidelines, white papers, reports, and other resources that can provide practical insights and recommendations from professional bodies and institutions. A combination of English and Korean keywords and subject headings related to health professions students, education, and cyberethics was employed on 25 January 2023. An acknowledged limitation of our search was the language constraint. Due to limited funding for translation services, searches in languages other than English or Korean were not feasible. This limitation might have excluded potentially relevant studies in other languages, possibly affecting the comprehensiveness of our review. The complete reproducible search strategy can be found in Table S1 (Supplementary Materials). 

### 2.3. Stage 3: Study Selection

#### 2.3.1. Eligibility Criteria

The inclusion criteria for studies in this scoping review were as follows: (1) participants had to be students in the health professions (e.g., nursing, medicine, physical therapy, physician assistant, occupational therapy, speech and language therapy, dental, dietetic, pharmacy); (2) studies had to describe aspects of cyberethics or internet ethics education (e.g., moral sensitivity, moral judgment, moral motivation, moral character); (3) studies had to take place in academic institutions (e.g., universities, colleges) in which cyberethics education programs were offered; and (4) all types of published and unpublished studies were considered, including primary research studies, systematic reviews, meta-analyses, letters, guidelines, websites, blogs, and conference abstracts or proceedings.

The following exclusion criteria were applied: (1) studies in which less than 50% of participants were health professions students, based on the majority rule; (2) studies including veterinary medicine students because our focus was primarily on human health professions, and the ethical issues in veterinary medicine might diverge from those in human health care; (3) studies involving health professions trainees, such as residents, fellows, and interns, because our study aimed to specifically focus on the foundational and formative stages of HPE. Trainees, due to their advanced stage and close proximity to full professional practice, often have experiences and challenges that differ from those of students in earlier stages of their education; (4) studies that focused on general medical or health ethics without specific relevance to cyberethics; (5) studies reporting phenomena that were either too broad or not directly related to cyberethics in HPE; (6) studies focusing on internet addiction or behavioral problems, as these are distinct topics not central to our focus on cyberethics; or (7) studies lacking sufficient data to draw meaningful conclusions. There were no geographic restrictions on the eligibility criteria. Studies published from 1990 to the present were considered because distance and online education began to grow in the late 1990s with the advancement of the internet [2]. Detailed inclusion and exclusion criteria can be found in Table 1.

#### 2.3.2. Selection Process

After removing duplicates (*n* = 495), all identified studies were imported into Covidence, an evidence synthesis management software [26]. To ensure consistency in the screening process, team members underwent a calibration exercise. This helped in aligning our understanding and interpretation of the eligibility criteria. The screening process began once a 75% or greater agreement was reached among the team members, and refinements to the eligibility criteria and definitions were made through regular team discussions. Two reviewers independently screened the titles and abstracts of 1976 database studies and grey literature documents. Any conflicts that arose were resolved by an independent third reviewer. During the full-text screening phase, the documents were reviewed independently by two reviewers again, and any conflicts were resolved by a third reviewer. For Korean literature, the review was conducted by native Korean-speaking authors and followed the same process. A total of 37 documents met the eligibility criteria and were included for final review. Although backwards and forwards citation tracking of the final included articles was initially planned, it was ultimately not conducted due to resource constraints and the comprehensive nature of the initial search. The team concurred that the extensive initial search yielded a substantial amount of relevant literature, including theses, websites, and other sources, obviating the need for additional citation tracking. A flowchart of the document selection process is presented in Figure S1 (Supplementary Materials).

### 2.4. Stage 4: Charting the Data

The data were extracted using a custom extraction tool developed by the researchers in Microsoft Word, which underwent a pilot phase for refinement. During the pilot stage, the research team reviewed the included variables and made iterative changes as needed. A single reviewer conducted the data extraction from each study. To minimize potential biases or errors associated with a single reviewer’s extraction, the extracted findings from both English and Korean literature were reviewed by an independent reviewer. This process served as a form of inter-rater reliability checking, ensuring consistency and accuracy in the data extraction. The review team resolved any cases of disagreement in the data by discussion. Each study was summarized based on the following categories: author(s), year, country, study objective(s), type of evidence, methodology, participants, definition or conceptualization of cyberethics, theoretical framework, summary of main ideas, study limitations and identified gaps, and cyberethics interventions (if applicable). For studies involving cyberethics interventions, the summary included details such as intervention description, setting, methods or resources used, evaluation tools, and key findings relevant to the research questions.

### 2.5. Stage 5: Collating, Summarizing, and Reporting the Results

The data analysis had three main objectives: (1) to describe how cyberethics has been defined and conceptualized in the literature; (2) to elucidate the tools, theories, models, or frameworks that have guided cyberethics education; and (3) to identify the methods used to teach cyberethics, including any interventions employed in the literature. Descriptive content analysis was employed by the reviewers to categorize and group qualitative and quantitative data as well as to identify research gaps. This involved an iterative process of coding the extracted data and grouping them into themes, patterns, and categories using researcher-created matrices in Microsoft Word (Version 2310). Quality appraisal was not conducted because the purpose of this scoping review was to provide a comprehensive overview of the breadth of knowledge in this area without explicitly considering study quality [23]. However, we acknowledge that the absence of quality appraisal might limit our ability to assess the strength of the evidence included in the review. This limitation potentially impacts the depth of insights derived from the presented studies.

## 3. Results

A total of 37 documents, including 35 English-language articles and 2 Korean-language studies, were selected for this scoping review, the majority (76%) of which were peer-reviewed articles and studies (*n* = 28), followed by grey literature (*n* = 9).

### 3.1. Sample Characteristics

Peer-reviewed literature included survey studies (*n* = 13), viewpoint/opinion pieces (*n* = 4), mixed methods studies (*n* = 3), qualitative studies (*n* = 3), intervention studies (*n* = 3), analysis (*n* = 1), and a narrative review (*n* = 1). The inclusion of a narrative review paper [27] on digital professionalism in HPE provided valuable context and highlighted shared concepts, issues, and gaps in the literature. This integration allowed for a comparative analysis that provided a fuller understanding of similarities, differences, and potential synergies between digital professionalism and cyberethics. Within the grey literature, document types included dissertations or master’s theses (*n* = 4), standards and resource guidelines (*n* = 3), a reporting tool (*n* = 1), and a blog (*n* = 1). One grey literature dissertation piece used an action research design with a cyberethics intervention [28].

Included documents (*n* = 37) were published within the period spanning 2012 through 2022, with the largest number originating from the United States (*n* = 17, 45.9%), followed by the United Kingdom (*n* = 4, 10.8%) and South Korea (*n* = 4, 10.8%). Detailed depictions of publication trends by country and year are provided in Figure S2 (Supplementary Materials). An examination of the subject matter revealed that a significant number of the documents were applicable to the nursing discipline (*n* = 14, 37.8%), followed by the dental (*n* = 12, 32.4%) and medical (*n* = 5, 13.5%) fields. Additionally, a mixture of health professions was represented (*n* = 4, 10.8%), including physician assistant (*n* = 1, 2.7%) and pharmacy (*n* = 1, 2.7%). The sample sizes of studies that incorporated human subjects varied notably, spanning from 11 to 880 participants. Furthermore, two studies undertook analyses involving non-human subjects, examining student-created social media accounts [29] and student-uploaded YouTube videos [30]. The demographic profile of participants, when reported, predominantly skewed toward female individuals of white ethnicity. Most of the studies predominantly engaged students as participants, with a select few also involving subsets of faculty members [28,31,32]. For more detailed information on the characteristics of the included studies, refer to Table S2 (Supplementary Materials).

### 3.2. Exploration of Digital Environments and Platforms

Regarding the exploration and discussion of digital environments and platforms within the context of the 37 included papers, three principal areas were discerned: (a) social media (*n* = 25, 67.6%), which included platforms such as Facebook, Twitter, and LinkedIn and involved scrutiny of patterns and participant interactions; (b) online learning (*n* = 7, 18.9%), which encompassed virtual academic programs, hybrid courses, electronic testing platforms, and online ethics tutorials; and (c) general cyberspace (*n* = 5, 13.5%), which encapsulated an assortment of unspecified cyber environments or amalgamations of diverse platforms.

Within the social media domain (*n* = 25), Facebook emerged as the platform most frequently referenced, trailed by YouTube, Twitter, and Instagram. Notably, researchers frequently dissected social media behaviors across multiple platforms rather than concentrating solely on a singular one. For instance, Kenny and Johnson [33] probed dental students’ perceptions across Facebook, Twitter, Instagram, and YouTube, and Henry and Molnar [29] spotlighted unprofessional content on Facebook. Several articles gauged social media usage patterns among health professions students, scrutinizing their attitudes toward online behaviors [32,33,34,35,36,37,38,39,40,41] and investigating ramifications of patient–student clinician relationships and privacy [42,43,44,45].

The online learning category primarily concentrated on issues of academic dishonesty and integrity within virtual education settings [28,46,47,48,49]. The general cyberspace category encompassed studies with unspecified cyber environments or a blend of several platforms (e.g., online forum and social media) [50]; these studies pertained to topics such as the protection of patient health information [51]; digital professionalism [27]; and cyberbullying and cyberethics awareness [5,52].

### 3.3. Conceptualization of Cyberethics Literature

The term “cyberethics” reflects the need for clarification within the literature. Although all the included documents referenced this concept, only two [5,52] explicitly offered descriptions of it. Mosalanejad et al. [52] defined cyberethics as the philosophical exploration of ethical behavior in interdisciplinary computer networks and its societal and individual impacts. Kim and Choi [5], conversely, adopted a definition from another source, characterizing cyberethics as a standardized system delineating the morality of behavior in cyberspace, with a focus on preserving intellectual freedom, expression, and privacy. Cyberethics was frequently either contextualized using associated terms such as e-professionalism [32,38,45,53,54,55], information ethics [51], digital professionalism [27,30,37,56], or academic integrity [28,46,47,48,49], or contextualized using terminology indicative of uncivil behaviors, such as cyberbullying, unethical social media use, academic dishonesty, or unprofessional online conduct (e.g., documenting illegal or illicit activity, posting patient identifiers, uploading explicit images). Notably, nine studies used varying iterations of Cain and Romanelli’s [57] definition of e-professionalism to inform their work [27,30,32,38,45,53,54,56,58].

Most of the documents reviewed did not explicitly reference specific theoretical frameworks or models for guiding cyberethics education or research, a finding that mirrors the broader landscape of cyberethics research underscored by an included narrative review in which only 2 of 11 studies were rooted in pedagogical theory [27]. Among the limited instances of theoretical framework use, the theory of planned behavior stood out, as evidenced in works by Cha [51] and Gormley et al. [37]; other instances included social cognitive theory, the theory of students cheating and plagiarism [48], systems theory, and change theory [28]. Notably, Kim and Choi [5] devised their own theoretical framework to synthesize predictors of cyberethics awareness among nursing students, and Lie and colleagues [40] formulated a theoretical framework based on their findings to guide educators in integrating cyberethics pedagogy into established curricula.

Although instances of explicit frameworks were limited, many articles and documents adhered to ethical principles (e.g., autonomy, beneficence, nonmaleficence, justice) and ethical behavior in health care rooted in diverse ethical frameworks and the constructivist paradigm, which posits individuals’ subjective experiences and perspectives as the basis of all knowledge [59]. Several articles drew guidance from professional organizations to inform their cyberethics work, including the American Medical Association (AMA) [40,60,61], the American Nurses Association (ANA) [44,55,62], the United Kingdom General Dental Council (GDC) [33,63], and the Nursing and Midwifery Code (NMC) [56,58]. Figure S3 (Supplementary Materials) presents a visual overview summarizing the conceptualization of cyberethics literature, illustrating key definitions, associated terms, theoretical frameworks, and references to professional organizations. For more detailed information on the conceptualization of cyberethics identified in the included studies, refer to Table S3 (Supplementary Materials).

### 3.4. Limitations, Gaps, and Recommendations from the Extracted Literature

Study limitations and identified gaps were extracted to gauge the replicability of findings and provide guidance for future research. The main limitations and gaps identified in the studies focused on the limited generalizability of findings, primarily due to factors such as convenience sampling, restricted sampling from a single academic institution or setting, and a lack of diversity in terms of gender and ethnicity among the study samples. Common recommendations from the authors of the included studies emphasized the need for institutions to establish policies and guidelines addressing cyberethics and to formally integrate related training into the curriculum.

### 3.5. Cyberethics Interventions and Outcomes

Within the included articles, four studies implemented cyberethics educational interventions [28,37,40,47]. These interventions were delivered through online or hybrid courses in two cases [28,47], and the mode of delivery was not specified in the other two studies [37,40]. The duration of interventions varied, for the times including 2 hours [37,40], one class day [47], and 3 weeks [28]. The target audience encompassed dental students (*n* = 2), a mix of health professions students (*n* = 1), and medical students (*n* = 1). The interventions focused on various aspects of cyberethics, such as online academic integrity (*n* = 2) and digital or social media professionalism (*n* = 2).

The evaluations of these interventions explored student attitudes, knowledge, and behaviors related to cyberethics. Azulay Chertok et al. [47] found that a face-to-face training module on online academic integrity and plagiarism significantly improved knowledge among health professions students compared to regular content review. Ellis [28] implemented an online integrity module for dental students and faculty which resulted in improved comprehension of academic honesty. Gormley et al. [37] reported a two-part intervention for dental students that combined an online professionalism seminar with personalized feedback based on an analysis of participants’ social media profiles; this intervention increased awareness of digital professionalism. Lie et al. [40] conducted a teaching session with medical students on social media and professionalism which led to thoughtful reflection and prompted participants to enhance their privacy settings. Comprehensive information on cyberethics interventions and outcomes can be found in Table 2.

## 4. Discussion

This scoping review aimed to comprehensively explore the pedagogy of cyberethics within HPE to provide a fuller understanding of the definitions and conceptualizations of cyberethics, the tools employed to guide cyberethics education, and the methods utilized in cyberethics interventions.

### 4.1. Definition and Conceptualization of Cyberethics

Our review underscored a significant gap in the explicit definition and conceptualization of cyberethics in the reviewed articles. Although only a few of the 37 articles reviewed provided explicit definitions, many alluded to varying terms and concepts. For instance, the term “e-professionalism” was used in some studies to refer to the ethical and professional behaviors expected of health professionals in the online space, while “cybercivility” was often associated with promoting respect and civility in digital interactions. “Digital professionalism” in other contexts emphasized the integration of traditional professional values with the evolving norms of digital interaction. Depending on context, others employed a variety of terms, such as “academic integrity in the online learning environment” or “patient-targeted googling (PTG).” This inconsistency in terminology and context-specific definitions could impede effective communication, hinder interdisciplinary dialogue, and obstruct a uniform understanding and application of ethical considerations associated with digital technologies in HPE.

### 4.2. Theoretical Frameworks

The majority of the studies we reviewed did not reference specific theoretical frameworks or established guidelines. This omission can lead to inconsistent practices across individuals and institutions. A previous scoping review on the pedagogical foundations of cybercivility education for health professions underscored the significance of several theoretical frameworks, such as transpersonal caring theory, media ecology communication, privacy management theory, and moral development theory [9]; these robust frameworks were largely overlooked in our sample. This omission could potentially lead to ethical gaps and diverse interpretations of ethical principles and guidelines related to digital technology use, resulting in technology misuse or other ethical lapses that could adversely affect students, educators, patients, and the broader community. Furthermore, the absence of explicit references to theoretical frameworks or models could (a) diminish the replicability of research and impede its further advancement, (b) obscure researchers’ understanding of the foundational assumptions, concepts, and relationships underpinning a study’s design and its authors’ interpretation of results, and (c) limit the ability to build upon or extend studies in subsequent research.

To address this issue, it is crucial to establish a clear definition of cyberethics and associated theoretical frameworks. Such efforts would facilitate the integration of ethical discussions and education into health professions curricula. A well-defined framework empowers students to effectively navigate ethical challenges in their professional practice, thus promoting desired outcomes such as positive behavioral manifestations, academic integrity, and digital professionalism [64]. For instance, feminist pedagogy could serve as a robust foundation for HPE as this approach prioritizes diverse learner perspectives, fosters self-reflection, challenges oppressive systems, encourages collaborative problem solving within the learning community, and ultimately advocates for justice and equity in an inclusive manner [65]. The media ecology theory also offers promise; by integrating ecological systems theory into cyberethics education [66], students can view cyberspace not as an isolated environment but as a complex, interconnected system requiring responsible and ethical behavior [9]. To foster rigor and progression in the field of cyberethics within HPE, researchers should transparently articulate and justify the theoretical frameworks or models informing their work. This endeavor lays the foundation for systematic and cumulative knowledge growth, enhancing the potential for replicating and extending research findings.

### 4.3. Outcomes of Interventions

Our review identified four intervention studies: two focused on academic integrity in online education and two focused on professional social media usage. These studies assessed outcomes related to students’ knowledge, attitudes, and both current and anticipated behaviors in the online environment. For example, following the intervention by Ellis [28], both dental students and faculty demonstrated an enhanced understanding of online academic integrity principles, as evidenced by post-intervention quizzes. Similarly, Gormley et al. [37] observed that, after their two-part intervention, dental students became more aware of the importance of digital professionalism, resulting in noticeable positive changes in their online presence. Furthermore, Azulay Chertok and colleagues [47] found their pre-posttest intervention positively influenced the attitudes of HPE students in both the control and intervention groups. Likewise, Lie et al. [40] reported increased professional role awareness among their sample of medical students. Although these studies reported promising results, many advised caution in interpreting the effectiveness of their interventions, pointing to limitations such as small sample sizes, sampling techniques, and methodological rigor. Additionally, these studies often centered on relatively narrow and indirect outcomes such as profile modifications, enhanced knowledge, or perceived motivation. Given the wide spectrum of online behaviors, future research should focus on tangible behavioral changes, both immediate and over time, as well as on practical implications.

### 4.4. Recommendtations for Future Research and Implications for Education

Based on our findings, the implications for practice, policy, and research in the field of cyberethics within HPE are significant.

#### 4.4.1. Definition and Conceptualization

Future research should invest more effort in clarifying the conceptualization of cyberethics and developing solid theoretical foundations. This endeavor could promote positive shifts in the actual online behaviors and attitudes of health professions students. Conducting a concept analysis of cyberethics in the context of health professions might be a beneficial avenue for future exploration. Prior studies have emphasized health professions students’ demand for comprehensive and practical guidelines to inform responsible online behaviors [20]. However, a review of 230 U.S. nursing schools’ policies and guidelines on proper behaviors in social media, online learning, and email writing revealed that only a third of these schools provided guidelines for their students, and fewer than 10% had guidelines for online classrooms and email usage [64]. Consistent with these findings, our review identified only a few studies suggesting cyberethics interventions or comprehensive guidelines within the realm of HPE.

It is also noteworthy that the majority of studies spotlight problematic situations without offering sufficient strategies to foster professional behaviors and desired outcomes in the swiftly evolving online environment. Although the samples in our review largely focused on traditional social media platforms such as Facebook, Twitter, Instagram, or blogs, a recent study highlighted the potential advantages and challenges of podcasting in nursing and midwifery education [67]. Similarly, concerns about platforms such as TikTok, which are popular among younger users but often overlooked in cyberethics discussions due to their perceived lack of professional implications, warrant attention [68]. The increasing prominence of tools such as ChatGPT has sparked discussions about potential ethical dilemmas and deception in nursing and other HPE [69,70]. Given the dynamic nature of digital spaces and associated technologies, educators must remain attuned to these advancements and continuously refine ethical guidelines. Moreover, innovative teaching strategies that nurture students’ values and motivations through deep self-exploration and reflection are essential to their success as future patient-centered health care professionals [1].

#### 4.4.2. Cultural Sensitivity

Culture differences should be carefully considered when developing cyberethics guidelines for HPE. As our review encompassed global samples, we acknowledge that cultural nuances can shape individuals’ values, beliefs, and ethical frameworks, influencing their perceptions and behaviors in the digital sphere. For instance, in certain cultures, the act of sharing patient details, especially with the eldest family member, might be seen as not just acceptable but also a sign of respect and trust. On the other hand, in other societies, this could be perceived as a serious breach of privacy and a violation of professional ethics. Such deeply ingrained cultural practices and beliefs can have profound implications on the expectations and standards set for online behaviors. Similarly, the concept of privacy and confidentiality can vary widely based on cultural contexts. In some societies, an open sharing of personal experiences, including health challenges, is encouraged as it fosters community support and collective understanding. In contrast, other cultures might prioritize individual privacy and discretion, considering such disclosures inappropriate or even taboo. These cultural variations also extend to online behaviors. As our review highlighted, the online behaviors, attitudes, and perceptions of South African nursing students [71] regarding online professionalism significantly differed from those of their counterparts in Malaysia and Indonesia [38]. Such disparities could be attributed to differences in cultural backgrounds, societal norms, and the emphasis on cyberethics in their respective educational systems.

#### 4.4.3. Implications for Education

In shaping cyberethics guidelines, it is imperative for educators to emphasize cultural sensitivity and inclusivity, ensuring applicability across diverse contexts. A universal approach might not cater to the unique nuances of different cultures. By incorporating insights from various cultural backgrounds, guidelines can achieve greater depth and relevance. To authentically incorporate cultural diversity, it is paramount to seek feedback from faculty and students from diverse backgrounds. Their unique experiences provide invaluable insights, instrumental in crafting inclusive and robust educational programs. As the digital landscape continually advances, with challenges especially pronounced in emerging countries [4], educators must remain updated. A focus on cultural sensitivity in cyberethics, coupled with a deep understanding of varied interpretations of essential concepts, grounded within time-tested theoretical frameworks, is pivotal for directing future research and best practices in HPE.

Furthermore, at the heart of cyberethics education lies the essential task of fostering students’ values and character development. Even as technological progress offers more efficient learning avenues, Whittier [72] highlighted the potential pitfalls of the digital realm. The asynchronous and impersonal facets of cyberspace can, at times, lead to users engaging with a sense of detachment, risking a decline in empathy. Such a digital milieu might hinder the discernment of vital social cues, which are paramount for ethical deliberation. Against this backdrop, Whittier [72] accentuated the need to anchor cyberethics education in the comprehensive nurturing of student character. Integrating fundamental virtues such as empathy, respect, honesty, responsibility, and trust becomes crucial in cultivating ethical digital citizens.

#### 4.4.4. Strengths and Limitations of the Study

The present scoping review has several notable strengths. First, our comprehensive search strategy, conducted by professional medical librarians, employed a range of databases and grey literature sources to ensure thorough coverage of the literature. This approach minimized the risk of missing relevant studies and enhanced the comprehensiveness of the review. Second, the screening and data extraction processes were conducted independently by multiple reviewers, with conflicts resolved through consensus discussions or third-party involvement. This rigorous approach improved the reliability and accuracy of the study selection and data extraction. Furthermore, the inclusion of both qualitative and quantitative studies, as well as grey literature, allowed for a thorough overview of the literature on cyberethics education for health professions students. This inclusive approach enriched understanding of the topic and increased the robustness of the findings.

Despite its strengths, this scoping review has limitations that should be acknowledged. One notable constraint was the linguistic scope of our review. Due to resource constraints, we focused solely on documents published in English and Korean. This introduces the potential for language bias, possibly excluding valuable insights from studies published in other languages. To address such limitations in future research endeavors, collaborations with multilingual researchers or incorporating reviewers fluent in various languages would be advantageous. While we made every effort to be thorough in our search, it is possible that some relevant documents may have evaded detection. The decision against conducting citation tracking of included articles, driven by resource limitations and the initial search’s exhaustive nature, heightens the risk of missing potentially relevant studies. A suggested remedy for future research endeavors would be to ensure periodic updates of the review and to invest in thorough citation tracking. It is worth noting that there might be studies, especially those discussing the intersection of cyberethics with emerging technologies such as generative artificial intelligence (AI), that this review might have inadvertently missed. The evident gap in discussions related to generative AI’s cyberethics in our review emphasizes the pressing need for more exploration in this area. Consequently, forthcoming research should prioritize a wider linguistic inclusion and actively delve into the ethical implications of emerging technologies, ensuring that the discourse remains relevant and timely in the rapidly evolving landscape of HPE.

## 5. Conclusions

In our scoping review, we uncover two central challenges within cyberethics education in health professions: the lack of explicit definition and robust conceptualization of cyberethics, and the absence of references to specific theoretical frameworks in the analyzed literature. These gaps underscore the need for a cohesive, universally accepted framework and a clear theoretical foundation to anchor ethical considerations in digital technology use. Our work emphasizes the criticality of incorporating cyberethics into health professions curricula to foster a consistent understanding and application of ethical principles. Furthermore, it calls for the creation of culturally sensitive and inclusive cyberethics guidelines to ensure relevance across diverse educational contexts. Although our review boasts comprehensive search methods and rigorous screening, future research should tackle limitations by encompassing studies in multiple languages, conducting comprehensive citation tracking, and exploring cyberethics in the context of emerging technologies such as generative AI. Addressing these identified gaps and needs will not only bolster cyberethics education, but also cultivate ethically sound digital practices, benefiting students, educators, patients, and the global community at large.

## Figures and Tables

**Table 1:** Eligibility Criteria.

	Inclusion	Exclusion
Population	Health professions students (e.g., nursing, medicine, physical therapy, physician assistant, occupational therapy, speech and language therapy, dental, dietetic, pharmacy)	Studies in which less than 50% of participants were comprised of health professions students Veterinary medicine students Health professions trainees (e.g., residents, fellows, interns)
Concept	Cyberethics or internet ethics education (e.g., moral sensitivity, moral judgment, moral motivation, moral character)	Studies reporting general medical or health ethics and not directly related to cyberethics Studies reporting a phenomenon too broad and/or not directly related to cyberethics in health professions education Studies focused on internet addiction or behavioral problems
Context	Academic institutions (e.g., universities or colleges) where cyberethics education programs are offered Worldwide (no geographical limits)	Nil
Types of sources of evidence	All types of published and unpublished studies including primary research studies, systematic reviews, meta-analyses, letters, guidelines, websites, blogs, conference abstracts or proceedings	Studies reporting insufficient data to draw meaningful conclusions

**Table 2:** Characteristics of Cyberethics Interventions (*n* = 4).

Author(s) (Year)	Aim/Design	Interventional Setting/Length	Intervention Methods/Process	Evaluation Method	Results
Azulay Chertok et al. (2014) [47]	Quasi-experimental study with a survey in a pre-posttest design study with an intervention to improve health sciences students’ knowledge and attitudes regarding academic integrity	Undergraduate health sciences hybrid courses; intervention delivered in person/ One class day	Intervention group received face-to-face oral presentation on online academic integrity and plagiarism based on the university’s policy Control group reviewed the policy on their own via the syllabus	Survey of online learning knowledge and attitudes (SOLKA) used to determine the baseline differences in scores between the control group and experimental group and the scores immediately following the intervention	No significant difference between control and intervention mean baseline knowledge scores or baseline attitude scores towards violation of academic integrity After intervention, both the control and intervention groups showed improved attitudes about academic integrity with a more significant knowledge increase in the intervention group
Ellis (2016) [28]	Mixed method action research study which used an academic tutorial module to increase dental students’ awareness of issues and consequences of academic dishonesty	Online community college dental program, students were enrolled in one of two courses: (1) Dental Radiography I (2) Infection Control/ Over a 3-week period in the 15-week semester	Within the tutorial, there were five online modules describing different aspects of academic integrity The online modules included discussions, online scavenger hunts, written assignments, and mini case studies	Quantitative evaluation tools: The academic integrity tutorial assessments and the student survey of online academic integrity Qualitative evaluation tools: Pre and post student surveys on online academic integrity, pre online faculty interviews, student feedback questionnaires, and academic integrity tutorial discussion posts	The implementation of the academic integrity tutorial improved academic honesty comprehension among online dental assisting students (88%) and faculty (100%) at a community college Both groups perceived the tutorial as engaging, thus increasing their motivation to increase knowledge
Gormley et al. (2021) [37]	Cross-sectional interventional study using focus groups to examine the impact of a “brownbag intervention” on behavior change regarding digital professionalism awareness	Dental school in the UK which delivers a professionalism program as a mandatory course during year 2 of undergraduate dental education/ 2.5 h	Two-part intervention: (1) One 2.5 h seminar (intro to professionalism) including a lecture from a guest speaker focused on e-professionalism (2) The “Brown Envelope Intervention” involved researchers creating Facebook profiles to review dental students’ publicly available social media information, which was summarized and given to each student in a sealed brown envelope during the seminar	Focus groups guided by a qualitative framework analysis and interview guide	Four main themes emerged: (1) Expression of student autonomy and any rejection of regulation, (2) Online activity in dentistry is different than medicine, (3) Intervention is useful and changed online behavior, (4) Constructive suggestions for training enhancement The ‘brown envelope’ intervention was well received and appears to prompt changes in student Facebook privacy settings
Lie et al. (2013) [40]	A mixed method intervention study aimed to increased awareness and action among students to change their online presence to reflect their emerging professional roles	Medical school that offers 200-h mandatory course called “Professionalism and the Practice of Medicine, (PPM)” that covers the 2011 American Medical Association (AMA) social media use guidelines 2 h	Training session called “Online Social Media and Professionalism,” within the 200 h mandatory course The session began with a large-group didactic presentation about the importance of reflection in practice, followed by a 30 min interactive lecture on maintaining a professional online presence using content from recent literature, media, and videos Then, students participated in an hour-long small-group discussion, led by faculty using a standardized script In small groups, students discussed their current online presence, social networking habits, professional implications of their behaviors, and examined a patient scenario	Written student reflections, course evaluations, and a four-month follow-up survey	Participation in the intervention resulted in reflection, increased professional role awareness, intention to change future online activities and monitor the activity of other medical students, which remained present at the follow-up evaluation Three domains emerged: (1) Immediate action (2) Intended future action, (3) Value change

## Data Availability

Not applicable.

## Supplementary Materials

The following supporting information can be downloaded at: https://www.mdpi.com/article/10.3390/ijerph20227048/s1, Table S1: Search results; Table S2: Characteristics of the included documents (*n* = 37); Table S3: Conceptualization of cyberethics identified in the included documents (*n* = 37); Figure S1: PRISMA flowchart; Figure S2: Publication trends by country and year; Figure S3: Conceptualization of cyberethics in the literature.
