# Supplementary material for: Exploration of Cyberethics in Health Professions Education: A Scoping Review
Source: Int J Environ Res Public Health. 2023 Nov 10;20(22):7048. doi: 10.3390/ijerph20227048 (PMC10671151; doi:10.3390/ijerph20227048)
Supplement: Supplementary file 1 [file ijerph-20-07048-s001.zip › Figure S1_PRISMA flowchart for the article search.pdf]

Figure S1. PRISMA-ScR (Preferred Reporting Items for Systematic Reviews and Meta-Analyses extension for Scoping Reviews) flow chart for the article search

(a) English article search flow chart

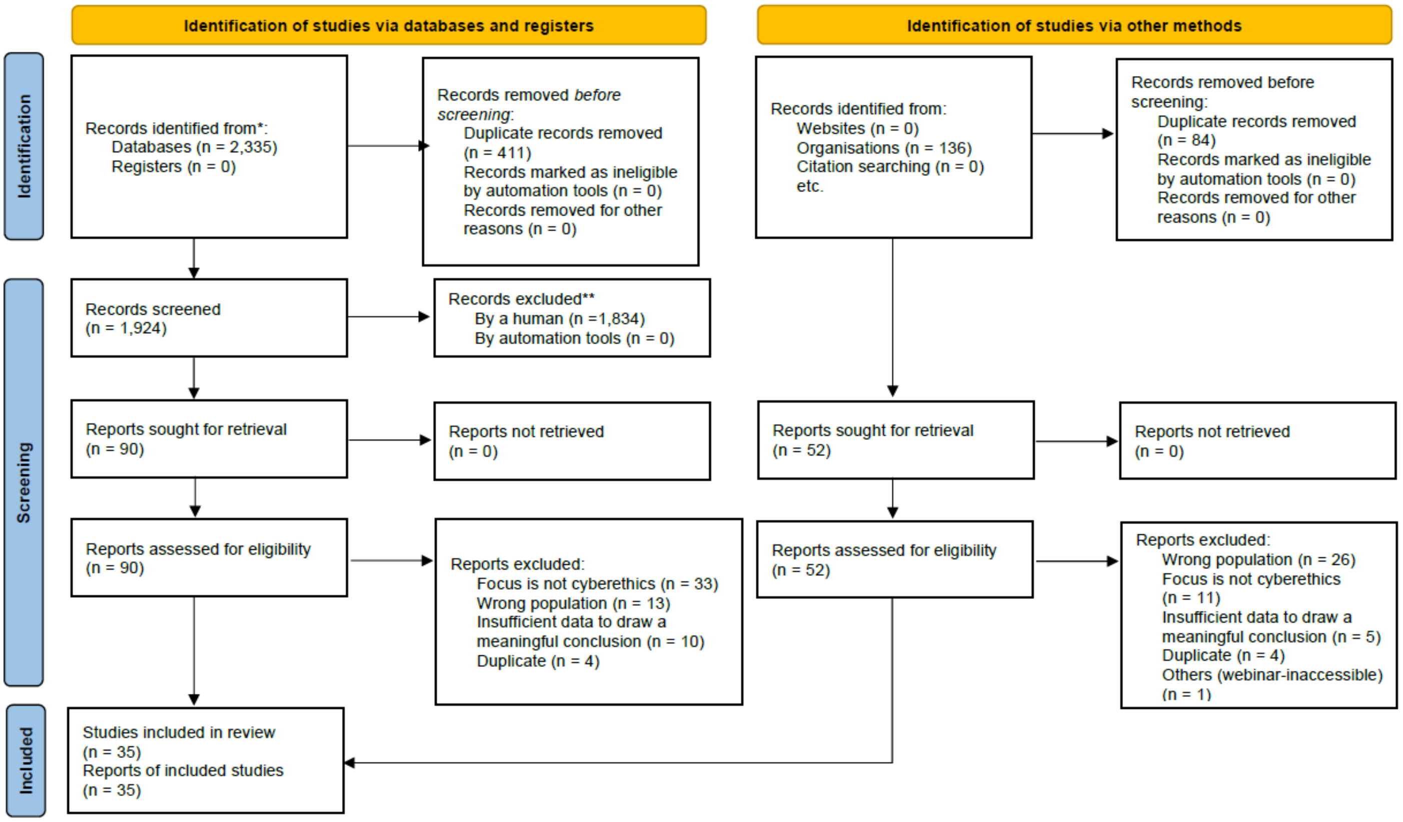

(b) Korean article search flow chart

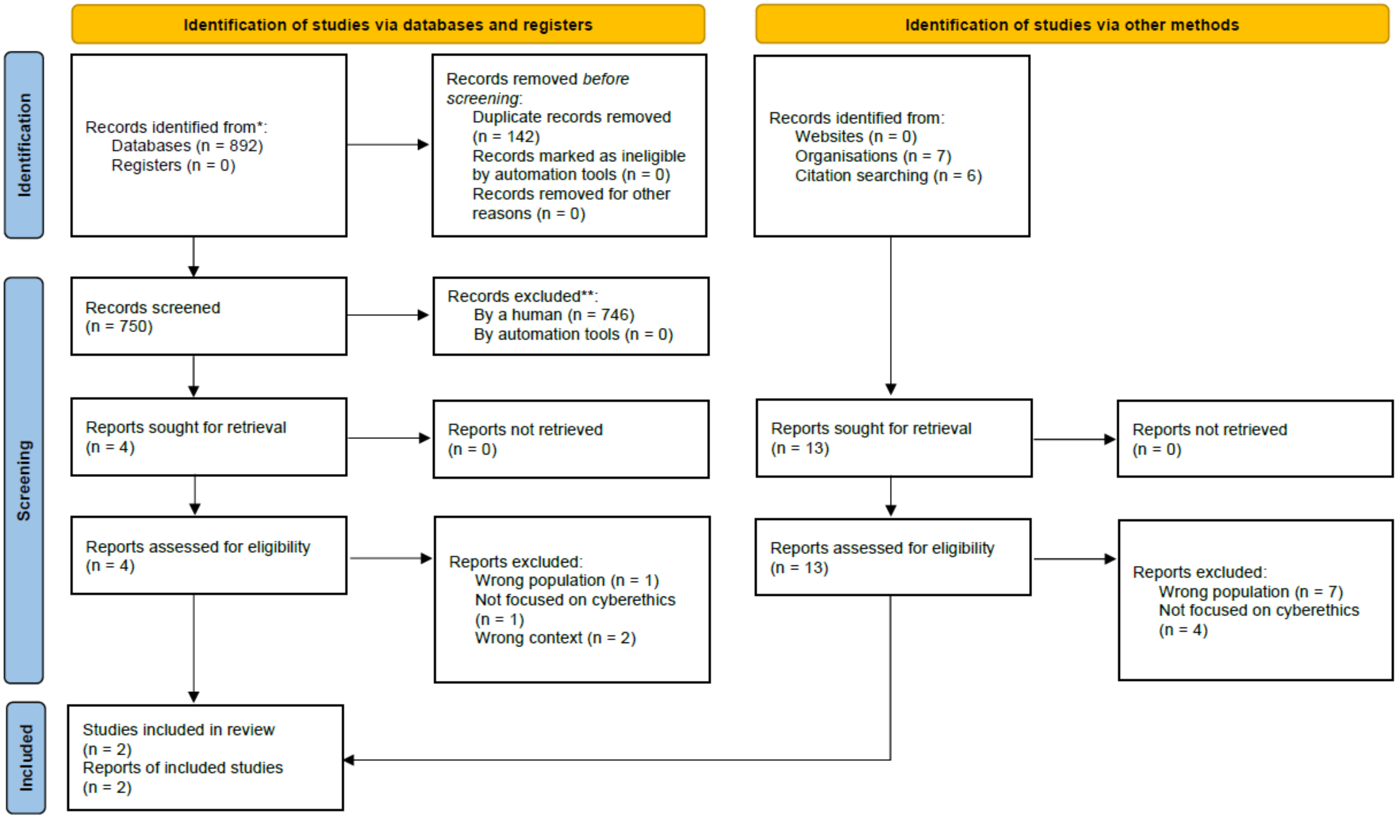

*Consider, if feasible to do so, reporting the number of records identified from each database or register searched (rather than the total number across all databases/registers).

**If automation tools were used, indicate how many records were excluded by a human and how many were excluded by automation tools.

From: Page MJ, McKenzie JE, Bossuyt PM, Boutron I, Hoffmann TC, Mulrow CD, et al. The PRISMA 2020 statement: an updated guideline for reporting systematic reviews. BMJ 2021;372:n71. doi: 10.1136/bmj.n71. For more information, visit: http://www.prisma-statement.org/
