# Supplementary material for: Exploration of Cyberethics in Health Professions Education: A Scoping Review
Source: Int J Environ Res Public Health. 2023 Nov 10;20(22):7048. doi: 10.3390/ijerph20227048 (PMC10671151; doi:10.3390/ijerph20227048)

Figure S2. Publication trends by country and year.

(a) Publication trends by country

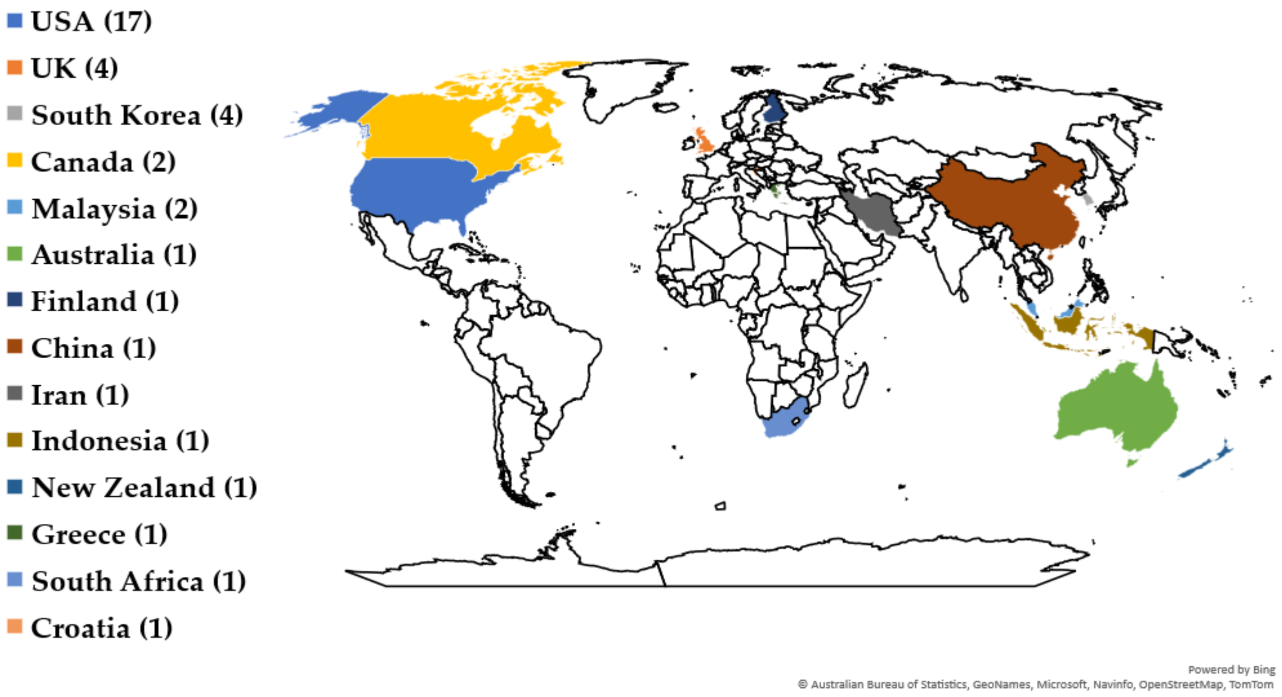

(b) Publication trends by year

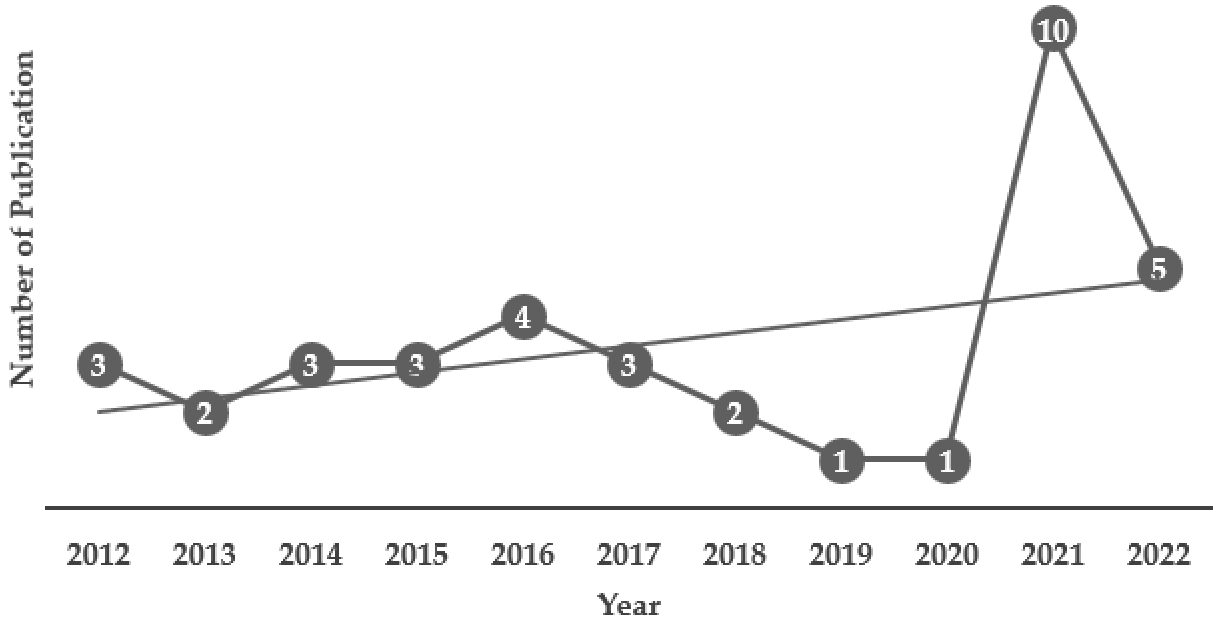

Supplement: Supplementary file 1 [file ijerph-20-07048-s001.zip › Figure S2_Publication trends by country and year.pdf]
