# Supplementary material for: Exploration of Cyberethics in Health Professions Education: A Scoping Review
Source: Int J Environ Res Public Health. 2023 Nov 10;20(22):7048. doi: 10.3390/ijerph20227048 (PMC10671151; doi:10.3390/ijerph20227048)
Supplement: Supplementary file 1 [file ijerph-20-07048-s001.zip › Table S1 Search Results .pdf]

Table S1: Search Results

Databases: Medline (via Ovid), CINAHL (via Ebscohost), PsycInfo (via Ebscohost), Sociology Source Ultimate (via Ebscohost), Sociology Source Ultimate (via Ebscohost), Proquest Dissertations & Theses Global

Date: 01/25/23
Searcher: Leila Ledbetter, MLIS, AHIP, Duke University Medical Center Library and Archives

Database: Medline (via Ovid)

Search	Query	Records retrieved
1<br>Health Professions Students/education	"Education, Professional"/ or exp "Education, Medical"/ or exp "Education, Nursing"/ or "Education, Graduate"/ or exp "Education, Dental"/ or exp "Education, Pharmacy"/ or exp "Education, Public Health Professional"/ or "Schools, Health Occupations"/ or exp "Schools, Medical"/ or exp "Schools, pharmacy"/ or exp "Schools, Dental"/ or exp "Schools, public health"/ or exp "Students, Health Occupations"/ or exp "Faculty, Nursing"/ or exp "Faculty, Medical"/ or exp "Faculty, Dental"/ or exp "Faculty, Nursing"/ or exp "Faculty, pharmacy"/ or exp "Schools, Nursing"/ or exp "Students, Public Health"/ or exp "Students, Pharmacy"/ or exp "Students, Medical"/ or exp "Students, Nursing"/ or exp "Students, Dental"/	371,380
2<br>Health professions	(Nursing or nurse or nurses or medical or dental or pharmacy or "physical therapy" or "occupational therapy" or "public health" or "allied health" or chiropractic or midwifery or podiatry or "physician assistant"or "health professions" or "health occupations").ti,ab.	2,390,490
3<br>education	exp "Education, Distance"/ or education.fs. or exp Learning/ or exp Teaching/ or exp Students/	865,679
4<br>Health professions education	2 and 3	194,788
5<br>education	(education OR Educating OR educated OR educates OR educational OR learn OR learning OR learned OR learns OR teach OR teaching OR taught OR teaches OR school OR schools OR schooling OR student OR students OR class OR classes OR curriculum OR course OR courses OR classroom OR classrooms OR faculty OR university OR universities or college Or colleges OR pedagogy OR pedagogies or instructor or instructors).ti,ab.	3,181,041
6<br>Health professions education	2 AND 5	668,979
7	1 or 4 or 6	895,438
8<br>cyber	Exp "Internet Use"/ or exp "Social Media"/	15,311
9<br>cyber	(cyber* or internet or online or web or digital).ti,ab.	561,514
10<br>ethics	Exp "Morals"/	181,318
11<br>ethics	(Moral* or ethic* or integrity or cyberethics or cybermorality or immoral* or impropriety or unethic* or norms OR mores).ti,ab.	375,957
12<br>morality	8 and 10	492
13	9 adj5 11	1,140
14	12 or 13	1,608
15	7 and 14	296
16	limit 15 to yr="1990 -Current"	296

Database: CINAHL (via Ebscohost)

Search	Query	Records retrieved
1<br>Health Professions Students/education	(MH "Education, Medical+") OR (MH "Education, Nursing+") OR (MH "Education, Graduate+") OR (MH "Education, Dental") OR (MH "Education, Pharmacy") OR (MH "Schools, Health Occupations+") OR (MH "Schools, Medical") OR (MH "Schools, Allied Health") OR (MH "Students, Health Occupations+") OR (MH "Faculty, Nursing") OR (MH	219,969

	"Faculty, Medical") OR (MH "Faculty, Dental") OR (MH "Schools, Nursing") OR (MH "Students, Pharmacy") OR (MH "Students, Medical") OR (MH "Students, Nursing+") OR (MH "Students, Dental")	
2<br>Health professions	TI (Nursing OR nurse OR nurses OR medical OR dental OR pharmacy OR "physical therapy" OR "occupational therapy" OR "public health" OR "allied health" OR chiropractic OR midwifery OR podiatry OR "physician assistant" OR "health professions" OR "health occupations") OR AB (Nursing OR nurse OR nurses OR medical OR dental OR pharmacy OR "physical therapy" OR "occupational therapy" OR "public health" OR "allied health" OR chiropractic OR midwifery OR podiatry OR "physician assistant" OR "health professions" OR "health occupations")	1,281,983
3<br>education	(MH "Online Education") OR (MH "Learning+") OR (MH "Teaching+") OR (MH "Students+")	491,847
4<br>Health professions education	S2 and S3	135,542
5<br>education	TI (education OR Educating OR educated OR educates OR educational OR learn OR learning OR learned OR learns OR teach OR teaching OR taught OR teaches OR school OR schools OR schooling OR student OR students OR class OR classes OR curriculum OR course OR courses OR classroom OR classrooms OR faculty OR university OR universities OR college OR colleges OR pedagogy OR pedagogies OR instructor OR instructors) OR AB (education OR Educating OR educated OR educates OR educational OR learn OR learning OR learned OR learns OR teach OR teaching OR taught OR teaches OR school OR schools OR schooling OR student OR students OR class OR classes OR curriculum OR course OR courses OR classroom OR classrooms OR faculty OR university OR universities OR college OR colleges OR pedagogy OR pedagogies OR instructor OR instructors)	1,127,612
6<br>Health professions education	S2 AND S5	376,609
7	S1 or S4 or S6	518,509
8<br>cyber	(MH "Social Media+") OR (MH "Internet+")	67,844
9<br>cyber	TI (cyber* OR internet OR online OR web OR digital) OR AB (cyber* OR internet OR online OR web OR digital)	233,066
10<br>ethics	MH "Morals+"	42,830
11<br>ethics	TI (Moral* OR ethic* OR integrity OR cyberethics OR cybermorality OR immoral* OR impropriety OR unethic* OR norms OR mores) OR AB (Moral* OR ethic* OR integrity OR cyberethics OR cybermorality OR immoral* OR impropriety OR unethic* OR norms OR mores)	1,124,002
12<br>morality	S8 and S10	1,119
13	S9 N5 S11	5,734
14	S12 or S13	6,808
15	S7 and S14	964
16	Unable to limit to 1990 because the oldest citation available in the search is 1993	964

Database: PsycInfo (via Ebscohost)

Search	Query	Records retrieved
1<br>Health Professions Students/education	DE "Medical Education" OR DE "Medical Internship" OR DE "Medical Residency" OR DE "Psychiatric Training" OR DE "Graduate Education" OR DE "Dental Education" OR DE "Nursing Education" OR DE "Nursing Students" OR DE "Medical Students" OR DE "Dental Students"	46,598
2<br>Health professions	TI (Nursing OR nurse OR nurses OR medical OR dental OR pharmacy OR "physical therapy" OR "occupational therapy" OR "public health" OR "allied health" OR chiropractic OR midwifery OR podiatry OR "physician assistant" OR "health professions" OR "health occupations") OR AB (Nursing OR nurse OR nurses OR medical OR dental OR pharmacy OR "physical therapy" OR "occupational therapy" OR "public health" OR "allied health" OR chiropractic OR midwifery OR podiatry OR "physician assistant" OR "health professions" OR "health occupations")	376,486

3<br>education	DE "Distance Education" OR DE "Electronic Learning" OR DE "Learning" OR DE "Teaching" OR DE "Students"	201,738
4<br>Health professions education	S2 and S3	11,180
5<br>education	TI (education OR Educating OR educated OR educates OR educational OR learn OR learning OR learned OR learns OR teach OR teaching OR taught OR teaches OR school OR schools OR schooling OR student OR students OR class OR classes OR curriculum OR course OR courses OR classroom OR classrooms OR faculty OR university OR universities OR college OR colleges OR pedagogy OR pedagogies OR instructor OR instructors) OR AB (education OR Educating OR educated OR educates OR educational OR learn OR learning OR learned OR learns OR teach OR teaching OR taught OR teaches OR school OR schools OR schooling OR student OR students OR class OR classes OR curriculum OR course OR courses OR classroom OR classrooms OR faculty OR university OR universities OR college OR colleges OR pedagogy OR pedagogies OR instructor OR instructors)	1,674,462
6<br>Health professions education	S2 AND S5	142,295
7	S1 or S4 or S6	156,149
8<br>cyber	DE "Social Media" OR DE "Internet Usage"	17,751
9<br>cyber	TI (cyber* OR internet OR online OR web OR digital) OR AB (cyber* OR internet OR online OR web OR digital)	203,018
10<br>ethics	DE "Morality"	26,513
11<br>ethics	TI (Moral* OR ethic* OR integrity OR cyberethics OR cybermorality OR immoral* OR impropriety OR unethic* OR norms OR mores) OR AB (Moral* OR ethic* OR integrity OR cyberethics OR cybermorality OR immoral* OR impropriety OR unethic* OR norms OR mores)	1,510,279
12<br>morality	S8 and S10	107
13	S9 N5 S11	10,335
14	S12 or S13	10,416
15	S7 and S14	430
16	Unable to limit to 1990 because the oldest citation available in the search is 1999	430

Database: Sociology Source Ultimate (via Ebscohost)

Search	Query	Records retrieved
1<br>Health Professions Students/education	DE "PROFESSIONAL education" OR DE "GRADUATE students" OR DE "UNIVERSITIES & colleges"	30,457
2<br>Health professions	TI (Nursing OR nurse OR nurses OR medical OR dental OR pharmacy OR "physical therapy" OR "occupational therapy" OR "public health" OR "allied health" OR chiropractic OR midwifery OR podiatry OR "physician assistant" OR "health professions" OR "health occupations") OR AB (Nursing OR nurse OR nurses OR medical OR dental OR pharmacy OR "physical therapy" OR "occupational therapy" OR "public health" OR "allied health" OR chiropractic OR midwifery OR podiatry OR "physician assistant" OR "health professions" OR "health occupations")	124,295
3<br>education	DE "DISTANCE education" OR DE "ONLINE education" or DE "EDUCATION" OR DE "LEARNING" OR DE "TEACHING" OR DE "STUDENTS"	98,355
4<br>Health professions education	S2 AND S3	2,456
5<br>education	TI (education OR Educating OR educated OR educates OR educational OR learn OR learning OR learned OR learns OR teach OR teaching OR taught OR teaches OR school OR schools OR schooling OR student OR students OR class OR classes OR curriculum OR course OR courses OR classroom OR classrooms OR faculty OR university OR universities OR college OR colleges OR pedagogy OR pedagogies OR instructor OR instructors) OR AB (education OR Educating OR educated OR educates OR educational OR learn OR learning OR learned OR learns OR teach OR teaching OR taught OR teaches OR school OR schools	694,128

	OR schooling OR student OR students OR class OR classes OR curriculum OR course OR courses OR classroom OR classrooms OR faculty OR university OR universities OR college OR colleges OR pedagogy OR pedagogies OR instructor OR instructors)	
6<br>Health professions education	S2 AND S5	35,589
7	S1 OR S4 OR S6	65,131
8<br>cyber	DE "INTERNET users" OR DE "INTERNET" OR DE "SOCIAL media"	14,798
9<br>cyber	TI (cyber* OR internet OR online OR web OR digital) OR AB (cyber* OR internet OR online OR web OR digital)	61,907
10<br>ethics	DE "MORAL attitudes" OR DE "ETHICS"	17,647
11<br>ethics	TI (Moral* OR ethic* OR integrity OR cyberethics OR cybermorality OR immoral* OR impropriety OR unethic* OR norms OR mores) OR AB (Moral* OR ethic* OR integrity OR cyberethics OR cybermorality OR immoral* OR impropriety OR unethic* OR norms OR mores)	528,068
12<br>morality	S8 and S10	202
13	S9 N5 S11	2,866
14	S12 or S13	3,023
15	S7 and S14	78
16	Limiters - Published Date: 19900101-20221231	77

Database: Education Full Text (via Ebscohost)

Search	Query	Records retrieved
1<br>Health Professions Students/education	DE "Professional education" OR DE "Medical education" OR DE "Nursing education" OR DE "Graduate nursing education" OR DE "Public health nursing education" OR DE "Graduate education" OR DE "Graduate nursing education" OR DE "Pharmacy education" OR DE "Public health education" OR DE "Public health education (Graduate)" OR DE "Public health education (Higher)" OR DE "Health occupations schools" OR DE "Medical school faculty" OR DE "Dental education" OR DE "Dental health education" OR DE "Dental faculty" OR DE "Nursing school faculty" OR DE "Nursing schools" OR DE "Pharmacy students" OR DE "Medical students" OR DE "Chiropractic students" OR DE "Medical school applicants" OR DE "Podiatry students" OR DE "Women medical students" OR DE "Nursing students" OR DE "Dental students"	66,266
2<br>Health professions	TI (Nursing OR nurse OR nurses OR medical OR dental OR pharmacy OR "physical therapy" OR "occupational therapy" OR "public health" OR "allied health" OR chiropractic OR midwifery OR podiatry OR "physician assistant" OR "health professions" OR "health occupations") OR AB (Nursing OR nurse OR nurses OR medical OR dental OR pharmacy OR "physical therapy" OR "occupational therapy" OR "public health" OR "allied health" OR chiropractic OR midwifery OR podiatry OR "physician assistant" OR "health professions" OR "health occupations")	134,483
3<br>education	DE "Distance education" OR DE "Education" OR DE "Learning" OR DE "Active learning" OR DE "Teaching" OR DE "Students" DE "Distance education students" OR DE "Dutch students" OR DE "Education students"	262,211
4<br>Health professions education	S2 AND S3	10,295
5<br>education	TI (education OR Educating OR educated OR educates OR educational OR learn OR learning OR learned OR learns OR teach OR teaching OR taught OR teaches OR school OR schools OR schooling OR student OR students OR class OR classes OR curriculum OR course OR courses OR classroom OR classrooms OR faculty OR university OR universities OR college OR colleges OR pedagogy OR pedagogies OR instructor OR instructors) OR AB (education OR Educating OR educated OR educates OR educational OR learn OR learning OR learned OR learns OR teach OR teaching OR taught OR teaches OR school OR schools OR schooling OR student OR students OR class OR classes OR curriculum OR course OR courses OR classroom OR classrooms OR faculty OR university OR universities OR college OR colleges OR pedagogy OR pedagogies OR instructor OR instructors)	1,967,029

6<br>Health professions education	S2 AND S5	82,792
7	S1 OR S4 OR S6	112,667
8<br>cyber	DE "Internet users" OR DE "Social media" OR DE "Online chat" OR	16,196
9<br>cyber	TI (cyber* OR internet OR online OR web OR digital) OR AB (cyber* OR internet OR online OR web OR digital)	265,070
10<br>ethics	DE "Ethical problems"	572
11<br>ethics	TI (Moral* OR ethic* OR integrity OR cyberethics OR cybermorality OR immoral* OR impropriety OR unethic* OR norms OR mores) OR AB (Moral* OR ethic* OR integrity OR cyberethics OR cybermorality OR immoral* OR impropriety OR unethic* OR norms OR mores)	127,728
12<br>morality	S8 and S10	6
13	S9 N5 S11	1,892
14	S12 OR S13	1,897
15	S7 AND S14	84
16	Unable to limit to 1990 because the oldest citation available in the search is 1997	84

Database: Proquest Dissertations & Theses Global

Search	Query	Records retrieved
1<br>Health professions	NOFT(Nursing OR nurse OR nurses OR medical OR dental OR pharmacy OR "physical therapy" OR "occupational therapy" OR "public health" OR "allied health" OR chiropractic OR midwifery OR podiatry OR "physician assistant" OR "health professions" OR "health occupations")	337,093
2<br>education	NOFT(education OR Educating OR educated OR educates OR educational OR learn OR learning OR learned OR learns OR teach OR teaching OR taught OR teaches OR school OR schools OR schooling OR student OR students OR class OR classes OR curriculum OR course OR courses OR classroom OR classrooms OR faculty OR university OR universities OR college OR colleges OR pedagogy OR pedagogies OR instructor OR instructors)	4,964,986
3<br>cyberethics	NOFT((cyber* OR internet OR online OR web OR digital) NEAR/5 (Moral* OR ethic* OR integrity OR cyberethics OR cybermorality OR immoral* OR impropriety OR unethic* OR norms OR mores))	11,794
4	1 AND 2 AND 3	486
5	Applied filters: 1990-2029	484

Databases (Korean): RISS, KISS, DBpia, ScienceON

Date: 02/15/2023
Searcher: L. Yoo through the Ewha Womans University Library

<table>
<tr><th colspan="3">KISS (via EWHA Library) https://kiss.kstudy.com/</th></tr>
<tr><th>Set #</th><th>Search Strategy</th><th>Results</th></tr>
<tr><td>1.
Participants</td><td>[논문명] "간호"|"의료"|"의대"|"의과대"|"물리 치료"|"작업 치료"|"언어 치료"|"치대"|"치과"|"영양학"|"식품 영양학"|"약대"|"약학"|"보건"|"보건 의료"|"의료 보건"
OR
[초록] "간호"|"의료"|"의대"|"의과대"|"물리 치료"|"작업 치료"|"언어 치료"|"치대"|"치과"|"영양학"|"식품 영양학"|"약대"|"약학"|"보건"|"보건 의료"|"의료 보건"</td><td>2,703</td></tr>
<tr><td rowspan="2">2.
Concept</td><td>2-1.
[논문명] "온라인"|"디지털"|"사이버"|"인터넷"|"정보"|"정보 통신"|"통신"|"소셜 미디어"|"SNS"
OR
[초록] "온라인"|"디지털"|"사이버"|"인터넷"|"정보"|"정보 통신"|"통신"|"소셜 미디어"|"SNS"</td><td>4,798</td></tr>
<tr><td>2-2.
[논문명] "윤리"|"에티켓"|"시민성"|"비시민성"|"프로페셔널리즘"|"전문직업성"|"전문직 윤리"|"가이드라인"|"지침"</td><td>24,237</td></tr>
</table>

<table>
<tr><td rowspan="2"></td><td>OR
[초록] “윤리"|"에티켓"|"시민성"|"비시민성"|"프로페셔널리즘"|"전문직업성"|"전문직 윤리"|"가이드라인"|"지침"</td><td></td></tr>
<tr><td>(#2-1 논문명 AND #2-2 논문명) OR (#2-1 초록 AND #2-2 초록)</td><td>165</td></tr>
<tr><td>3.
Combine</td><td>(#1 논문명 AND #2 논문명) OR (#1 초록 AND #2 초록)</td><td>6</td></tr>
<tr><td>4.
NOT</td><td>"청소년"|"초등"|"중등"|"중학"|"고등"|"십대"|"10 대"

("간호"|"의료"|"의대"|"의과대"|"물리 치료"|"작업 치료"|"언어 치료"|"치대"|"치과"|"영양학"|"식품 영양학"|"약대"|"약학"|"보건"|"보건 의료"|"의료 보건")
("온라인"|"디지털"|"사이버"|"인터넷"|"정보"|"정보 통신"|"통신"|"소셜 미디어"|"SNS")
("윤리"|"에티켓"|"시민성"|"비시민성"|"프로페셔널리즘"|"전문직업성"|"전문직 윤리"|"가이드라인"|"지침") !("청소년"|"초등"|"중등"|"중학"|"고등"|"십대"|"10 대")</td><td>3</td></tr>
<tr><td>5.
Publication Year</td><td>1990 년 이전 제외</td><td>3</td></tr>
</table>

<table>
<tr><th colspan="3">DBpia (via EWHA Library) https://kiss.kstudy.com/</th></tr>
<tr><th>Set #</th><th>Search Strategy</th><th>Results</th></tr>
<tr><td>1.
Participants</td><td>[전체] "간호"|"의료"|"의대"|"의과대"|"물리 치료"|"작업 치료"|"언어 치료"|"치대"|"치과"|"영양학"|"식품 영양학"|"약대"|"약학"|"보건"|"보건 의료"|"의료 보건"<s>|"대학생"|"교육"</s></td><td>185,077</td></tr>
<tr><td rowspan="3">2.
Concept</td><td>2-1.
[전체] "온라인"|"디지털"|"사이버"|"인터넷"|"정보"|"정보 통신"|"통신"|"소셜 미디어"|"SNS"</td><td>185,077</td></tr>
<tr><td>2-2.
[전체] "윤리"|"에티켓"|"시민성"|"비시민성"|"프로페셔널리즘"|"전문직업성"|"전문직 윤리"|"가이드라인"|"지침"</td><td>81,935</td></tr>
<tr><td>(#2-1 AND #2-2)</td><td>15,436</td></tr>
<tr><td>3.
Combine</td><td>(#1 AND #2)</td><td>474</td></tr>
<tr><td>4.
NOT</td><td>"청소년"|"초등"|"중등"|"중학"|"고등"|"십대"|"10 대"

("간호"|"의료"|"의대"|"의과대"|"물리 치료"|"작업 치료"|"언어 치료"|"치대"|"치과"|"영양학"|"식품 영양학"|"약대"|"약학"|"보건"|"보건 의료"|"의료 보건")
("온라인"|"디지털"|"사이버"|"인터넷"|"정보"|"정보 통신"|"통신"|"소셜 미디어"|"SNS")
("윤리"|"에티켓"|"시민성"|"비시민성"|"프로페셔널리즘"|"전문직업성"|"전문직 윤리"|"가이드라인"|"지침") !("청소년"|"초등"|"중등"|"중학"|"고등"|"십대"|"10 대")</td><td>440</td></tr>
<tr><td>5.
Publication Year</td><td>1990 년 이전 제외</td><td>440</td></tr>
</table>

<table>
<tr><th colspan="3">RISS (via EWHA Library)</th></tr>
<tr><th>Set #</th><th>Search Strategy</th><th>Results</th></tr>
<tr><td>1.
Participants</td><td>[논문명] "간호"|"의료"|"의대"|"의과대"|"물리 치료"|"작업 치료"|"언어 치료"|"치대"|"치과"|"영양학"|"식품 영양학"|"약대"|"약학"|"보건"|"보건 의료"|"의료 보건"<s>|"대학생"|"교육"</s>
OR
[초록] "간호"|"의료"|"의대"|"의과대"|"물리 치료"|"작업 치료"|"언어 치료"|"치대"|"치과"|"영양학"|"식품 영양학"|"약대"|"약학"|"보건"|"보건 의료"|"의료 보건"<s>|"대학생"|"교육"</s></td><td>89,944</td></tr>
<tr><td rowspan="2">2.
Concept</td><td>2-1.
[논문명] "온라인"|"디지털"|"사이버"|"인터넷"|"정보"|"정보 통신"|"통신"|"소셜 미디어"|"SNS"
OR
[초록] "온라인"|"디지털"|"사이버"|"인터넷"|"정보"|"정보 통신"|"통신"|"소셜 미디어"|"SNS"</td><td>357,538</td></tr>
<tr><td>2-2.</td><td>58,636</td></tr>
</table>

	[논문명] "윤리"|"에티켓"|"시민성"|"비시민성"|"프로페셔널리즘"|"전문직업성"|"전문직 윤리"|"가이드라인"|"지침"<br>**OR**<br>[초록] "윤리"|"에티켓"|"시민성"|"비시민성"|"프로페셔널리즘"|"전문직업성"|"전문직 윤리"|"가이드라인"|"지침"	
	(#2-1 논문명 **AND** #2-2 논문명) **OR** (#2-1 초록 **AND** #2-2 초록)	165
3.<br>Combine	(#1 논문명 **AND** #2 논문명) **OR** (#1 초록 **AND** #2 초록)	652
4.<br>NOT	"청소년"|"초등"|"중등"|"중학"|"고등"|"십대"|"10 대"<br><br>("간호"|"의료"|"의대"|"의과대"|"물리 치료"|"작업 치료"|"언어 치료"|"치대"|"치과"|"영양학"|"식품 영양학"|"약대"|"약학"|"보건"|"보건 의료"|"의료 보건")<br>("온라인"|"디지털"|"사이버"|"인터넷"|"정보"|"정보 통신"|"통신"|"소셜 미디어"|"SNS")<br>("윤리"|"에티켓"|"시민성"|"비시민성"|"프로페셔널리즘"|"전문직업성"|"전문직 윤리"|"가이드라인"|"지침") !("청소년"|"초등"|"중등"|"중학"|"고등"|"십대"|"10 대")	24
5.<br>Publication Year	1990 년 이전 제외	24

**SCIENCE ON (via EWHA Library) https://scienceon.kisti.re.kr/main/mainForm.do**		
**Set #**	**Search Strategy**	**Results**
1.<br>Participants	[전체] "간호"OR"의료"OR"의대"OR"의과대"OR"물리 치료"OR"작업 치료"OR"언어 치료"OR"치대"OR"치과"OR"영양학"OR"식품 영양학"OR"약대"OR"약학"OR"보건"OR"보건 의료"OR"의료 보건"~~OR"대학생"OR"교육"~~	179,634
2.<br>Concept	2-1.<br>[전체] "온라인"OR"디지털"OR"사이버"OR"인터넷"OR"정보"OR"정보 통신"OR"통신"OR"소셜 미디어"OR"SNS"	377,477
	2-2.<br>[전체] "윤리"OR"에티켓"OR"시민성"OR"비시민성"OR"프로페셔널리즘"OR"전문직업성"OR"전문직 윤리"OR"가이드라인"OR"지침"	37,946
	(#2-1 전체) **AND** (#2-2 전체)	6,145
3.<br>Combine	(#1 전체) **AND** (#2 전체)	443
4.<br>NOT	"청소년"OR"초등"OR"중등"OR"중학"OR"고등"OR"십대"OR"10 대"<br><br>{("간호"OR"의료"OR"의대"OR"의과대"OR"물리 치료"OR"작업 치료"OR"언어 치료"OR"치대"OR"치과"OR"영양학"OR"식품 영양학"OR"약대"OR"약학"OR"보건"OR"보건 의료"OR"의료 보건") AND !("청소년"OR"초등"OR"중등"OR"중학"OR"고등"OR"십대"OR"10 대")} AND {("온라인"OR"디지털"OR"사이버"OR"인터넷"OR"정보"OR"정보 통신"OR"통신"OR"소셜 미디어"OR"SNS") AND !("청소년"OR"초등"OR"중등"OR"중학"OR"고등"OR"십대"OR"10 대")} AND {("윤리"OR"에티켓"OR"시민성"OR"비시민성"OR"프로페셔널리즘"OR"전문직업성"OR"전문직 윤리"OR"가이드라인"OR"지침") AND !("청소년"OR"초등"OR"중등"OR"중학"OR"고등"OR"십대"OR"10 대")}	425
5.<br>Publication Year	1990 년 이전 제외	425
