# Supplementary material for: Exploration of Cyberethics in Health Professions Education: A Scoping Review
Source: Int J Environ Res Public Health. 2023 Nov 10;20(22):7048. doi: 10.3390/ijerph20227048 (PMC10671151; doi:10.3390/ijerph20227048)
Supplement: Supplementary file 1 [file ijerph-20-07048-s001.zip › Table S2_Characteristics of the included documents.pdf]

Table S2. Characteristics of the included documents (*n* = 37).

Author(s) (Year) /Country	Document Objective(s)	Participants/ Setting	Evaluation Tools	Key Findings	Educational Implications
American Association of Colleges of Pharmacy (AACP) (2022) /USA [34]	To promote pharmacy educators' and students' comprehension of the advantages, risks, and ethical considerations associated with social media use, while nurturing professionalism and upholding the profession's reputation	N/A	Mixed method surveys informed the creation of the resource guide	While numerous pharmacy school students, faculty, and administrators lack social media policies and training, those with established policies perceive them as comprehensive; thus, a need exists for guidance on addressing policy violations.	Colleges and schools of pharmacy should: (1) develop professionalism and social media guidelines for students, faculty, and staff; (2) create a climate that fosters open inquiry, free expression, and academic freedom; (3) train and educate students and faculty on social media use that aligns with institutional and professional expectations.
Aplin-Snider et al. (2021) /USA [46]	To examine the perceptions of graduate nurse practitioner (NP) students regarding academic dishonesty in online testing	Public online NP program specializing in family health and geriatric acute care<br><br>n = 58; n = 33 Masters of Nursing (MSN); n = 25 Doctor of Nursing Practice (DNP)	Academic integrity survey with Likert style questions and one open-ended question	• Most students reported that holding test integrity was important to them and that they believed faculty and administrators need to hold high online exam integrity expectations for students.<br>• Three themes from the open-ended question: (1) Ethics (2) Deterrents (3) Integrity.<br>• First year MSN students trusted academic integrity processes more than third year DNP students.	Nurse educators should seamlessly integrate and strengthen the promotion of academic integrity throughout the entirety of the program, particularly as students advance, and they can utilize these insights to formulate educational initiatives underscoring the significance of academic integrity in nursing practice.
Azulay Chertok et al. (2014) /USA [47]	To evaluate the effectiveness of the educational intervention to increase students' knowledge and improve attitudes about academic integrity, as it relates to the online learning environment	Undergraduate health sciences students in hybrid courses n = 355; nursing (n = 94), pre-medical (n = 277), and exercise physiology, and other students (n = 34)	The survey of online learning knowledge and attitudes (SOLKA)	• Almost all students use the internet for academic work, with 62.4% regularly reviewing integrity statements.<br>• Academic integrity attitude correlates significantly with knowledge, and greater. review of integrity statements improves both.<br>• Female students and nursing students initially demonstrate better attitudes and knowledge about academic integrity.	Institutions should develop explicit guidelines encompassing academic integrity, incorporating measures against plagiarism and cheating, while also ensuring that faculty members are prepared to address ethical dilemmas related to cyberethics; fostering education on online academic integrity can further enrich students' comprehension, cultivate

		n = 161 in control group; n = 194 in intervention group		• After interventions, both the control and intervention groups showed improved attitudes about academic integrity with a more significant knowledge increase in the intervention group.	constructive perspectives, and mitigate instances of academic misconduct.
Barlow et al. (2015) /Australia [35]	To analyze medical students' social media usage patterns, identify factors linked to unprofessional content posting, and examine if exposure to social media professionalism guidelines reduces such behaviors	Medical students from 21 medical schools<br><br>n = 880	An online survey with a skip-logic design	• Most medical students use social media and 1/3 have posted unprofessional content.<br>• No association between knowledge of social media guidelines and unprofessional behavior.<br>• Unprofessional content was associated with alcohol use, racist content, and planning to change profile name to conceal identity.	Integrating social media education directly into the curriculum rather than solely offering guidelines should be a formal approach for faculty; while privacy settings can restrict the audience, they may not effectively diminish inappropriate content; thus, the integration of social media ethics into medical school curricula becomes imperative.
Burlington (2020) /Canada [48]	To explore student perceptions of academic integrity at an online nursing college	Second year psychiatric nursing students in an online program<br><br>n= 17 (approximate)	Semi-structured interviews and online survey tool	• Four main themes emerged (1) Deficit American Psychological Association (APA) knowledge, (2) Assignment instructions and academic writing in the curriculum, (3) Frustrations unique to learning online.<br>• Nursing students had a high rate of plagiarism and student perceptions of academic integrity informed their decision to plagiarize.	Initiating initiatives targeting the reduction of plagiarism and enhancement of academic integrity within educational institutions could yield benefits, underscoring the requirement for tailored and all-encompassing education to effectively mitigate instances of academic integrity violations.
Cha (2017) /South Korea [51]	To examine how information ethics impacts nursing students' awareness of medical information protection in their practice	Undergraduate nursing students with than one year of clinical experience from four nursing schools<br><br>n = 238	Structured self-report questionnaire	• Students with a strong understanding of information ethics and awareness of protecting medical information were more likely to practice medical information protection.<br>• Those holding religious beliefs and respect for others exhibit a higher rate of practicing medical information protection, indicating the influence of personal values on ethical conduct in the digital space.	Enhancing awareness regarding the consequences of unethical conduct in online environments and furnishing explicit ethical directives can facilitate improved practices among nursing students; educators should evaluate nursing students' grasp of information ethics prior to their practical training in clinical settings.

Chester et al. (2017) /New Zealand [42]	To address the lack of data and knowledge regarding patient-targeted googling (PTG), while also exploring the attitudes and motivation driving medical students' engagement in PTG	Final year medical students<br><br>n = 54 survey<br>n = 10 focus group	Electronic survey<br><br>Semi-structured focus groups	• Less than 20% of students admitted to conducting PTG; most students had negative attitudes towards PTG.<br>• High social media increased the likelihood of conducting PTG.<br>• Students requested explicit guidelines on PTG due to concerns over patient confidentiality and the reliability of online information.	Providing medical students with instruction on PTG and offering guidelines for its professional use is essential; while personal ethical values can influence healthcare professionals' PTG practices, additional education has the potential to foster a more standardized approach.
Clark et al. (2012) /USA [31]	To explore faculty and student perceptions of cyber-bullying and incivility in online learning, and discern strategies for both prevention and intervention in relation to these behaviors	Online baccalaureate nursing completion program n = 152 nursing students; n = 19 faculty	Incivility in Online Learning Environments (IOLE) survey	• Faculty and students perceived incivility to be a mild to moderate problem and students were more likely than faculty to report incivility.<br>• Faculty experienced uncivil behaviors by students (e.g., failure to complete assignment) and fellow faculty (e.g., belittling).<br>• Students reported uncivil treatment by faculty (e.g., threatening comments) and fellow students (e.g., making racial or ethnic slurs).	Fostering a respectful teaching-learning environment through the establishment of co-created norms by both faculty and students is vital for the success of online learning; addressing ambiguity and providing clear communication to reduce anxiety among online learners can effectively curb uncivil behaviors arising from fear, uncertainty, and distrust.
Commission on Collegiate Nursing Education (CCNE) (2021) /USA [43]	To describe the CCNE's standards and key elements for accrediting entry-to-practice nurse residency programs, and guidelines for evaluating these programs	N/A	N/A	Adherence to CCNE guidelines in designing and implementing nurse residency programs can foster good cyberethics among nurse educators and leaders.	Nurse residency programs should expand nurse residents' knowledge and skills related to safe patient care using technology, including telehealth, virtual health, and use of electronic health record (EHR).
de Peralta et al. (2019) /USA [60]	To provide guidance to dental educators and students about the possible advantages and drawbacks of utilizing social media in dental education, while promoting conscientious and prudent social media usage	N/A	N/A	• Social media can enhance dental student learning and communication among peers, faculty, and patients, supporting its use in dental education.<br>• In contrast, there are potential risks to social media use such as privacy violations, internet addiction, and creation of a negative digital footprint.	While educators should exercise caution concerning social media's capacity for distractions within the learning environment, it is noteworthy that despite the limited availability of comprehensive research, advantages of incorporating social media into dental education do exist.

Duke et al. (2017) /Canada [32]	To explore faculty and student use of social media in nursing education and the associated implications	Bachelor of Nursing program<br><br>n = 337 students<br>n = 29 faculty	Mixed method survey with Likert style, multiple choice, and open-ended questions	• Both students and faculty use social media for educational purposes, yet students use it more than faculty.<br>• While both students and faculty report use privacy features and knowledge of ethical implications, both groups had a gap in e-professionalism awareness.	Equipping both students and faculty with e-professionalism education is essential to uphold the professional standards of nursing conduct; enhancing faculty's familiarity with various social media platforms is equally important for effectively teaching e-professionalism.
Eichler (2018) /USA [36]	To provide a snapshot of the American Dental Association (ADA) guidelines for dental students to present themselves on social media professionally	N/A	N/A	• The ADA provides directives for staff and volunteers regarding social media usage, highlighting the crucial nature of maintaining a professional online presence as a dental student to enhance career progression and safeguard their professional identity.	Dental students and professionals can display cyberethics and promote a culture of responsible and ethical behavior in the digital world by following these guidelines.
Ellis (2016) /USA [28]	To determine effectiveness of an academic integrity tutorial to improve dental students' and faculty comprehension and experiences of academic integrity	Freshman dental assisting students and faculty in online courses<br><br>n = 16 students<br>n = 5 faculty	Survey tool, interviews, feedback, and discussion posts	• The intervention tutorial improved academic honesty comprehension among online dental assisting students (88%) and faculty (100%).<br>• Both faculty and students perceived the tutorial as engaging and motivating.<br>• Students revealed that academic integrity is a commitment, and most were aware of their institution's integrity policy.	School administrators should promote academic integrity by allocating time for academic integrity training as part of student and faculty orientation; it's important to recognize that students may not fully comprehend policies solely through reading course syllabi, and faculty should not make such assumptions.
Englund et al. (2012) /USA [44]	To explore undergraduate nursing students' awareness of ethical problems manifested through posting clinical information on social media sites	n = 125 nursing students; n=69 sophomores; n= 59 seniors	Author-developed survey based on the American Nurses Association (ANA) Code of Ethics	• Nearly all nursing students use social media, of which 70% were aware of their college's social media policy.<br>• Senior students had greater social media awareness.<br>• Both groups reported uncertainty about the appropriateness of posting online during clinical breaks and adequacy of facility staffing.	Nurturing the essential skills and values of proficiently utilizing social media with sustained professional accountability necessitates faculty guidance; nurse educators bear the responsibility of imparting the competencies and ethical principles requisite for adeptly navigating the digital landscape.
Gormley et al. (2021)	To describe the impact of digital professionalism	Dental students in years two through	Focus groups	• Four main themes emerged: (1) Expression of student autonomy and	While the collection of online social media profile data is relatively low

/UK [37]	awareness training provided at one UK-based institution	five<br><br>n = 11		any rejection of regulation, (2) Online activity in dentistry is different than medicine, (3) Intervention is useful and changed online behavior, (4) Constructive suggestions for training enhancement.<br>• An e-professionalism intervention can foster digital professionalism awareness.<br>• The 'brown envelope' intervention was well-received and appears to prompt changes in student Facebook privacy settings.	in resource intensity, it nevertheless demands time, effort, and modest administrative expenses; countering these resource constraints necessitates faculty backing and support.
Henry & Molnar (2013) /USA [29]	To examine the accessibility, amount, and type of unprofessional information on the Facebook (FB) profiles of dental hygiene and dental students	College of dentistry<br><br>n = 305 FB profiles; n = 258 dental students; n = 47 dental hygiene students	FB profiles analyzed quantitatively and qualitatively for publicly available inappropriate content	• More than half of students had accessible FB profiles.<br>• Unprofessional content was found on 5.8% of profiles, most commonly by second year students.<br>• Privacy violations included pictures of patients in the dental clinic and tagging a patient in a post.	Dental education institutions should create and enforce policies that bring awareness to professional social media use as well as incorporate this content into ethics courses.
Kamarudin et al. (2022) /Malaysia, Indonesia [38]	To describe the self-reported pattern of social media use, online professional behaviors, and perceptions toward e-professionalism among dental students at two universities	Two Southeast Asian Universities<br><br>n = 300; n = 150 from Malaysian University; n = 150 from Indonesian University	Electronic survey	• All students use Facebook and Instagram for socialization and academics.<br>• Both groups shared positive perceptions toward online professionalism statements and were concerned over unprofessional posts.<br>• Despite the majority of students claiming to be aware of professional guidelines on social media use, a significant number of students reported engaging in unprofessional behaviors online.<br>• Malaysian students had significantly better scores in terms of their professionalism behaviors compared to Indonesian students.	Recognizing disparities between dental students' online apprehensions and actual behaviors, the need for well-defined guidelines and training to cultivate e-professionalism values becomes evident; given the dynamic landscape of e-professionalism, educators play a pivotal role in formulating distinct social media directives for dental students to foster consciousness and professional digital behaviors.

Karveleas et al. 2021 /Greece [45]	To determine whether Greek dental students post unprofessional content on Facebook, if and how they interact with patients via Facebook, and whether they are aware of the impact of Facebook behavior on e-professionalism	Undergraduate dental students<br><br>n = 512; n = 119 first year; n = 91 second year; n = 125 fourth year; n = 95 fifth year	Multiple choice questionnaire	• Most respondents utilized Facebook for both entertainment and education with strict privacy settings.<br>• Unprofessional content and negative remarks about faculty were commonly posted, and a considerable number of students engaged with patients.<br>• While many students acknowledged the potential impact of their online presence on patients' perceptions, awareness of potential legal consequences for social media behavior was low.	There is an urgent need for e-professionalism to become part of the dental school curriculum, especially during the first years (one and two) before students enter clinical.
Keating (2016) /USA [39]	To provide social media guidelines for medical students and physicians	N/A	N/A	• Social media guidelines can promote responsible, professional, accountable online behavior while adhering to principles of integrity, honor, acceptance of diversity and commitment to ethical conduct.	Medical students and physicians can demonstrate adherence to cyberethics principles by following set guidelines.
Kenny & Johnson (2016) /UK [33]	To examine dental students' attitudes toward professional behavior on social media while determining the scope and manner of social media utilization and exposure to potential instances of unprofessional behavior	Undergraduate dental students<br><br>n = 155	Questionnaire	• Most students use social media, primarily Facebook, with privacy controls in place and do not believe social media behavior should be separate from dental school.<br>• Most students reported awareness of professional guidelines and taking action to promote ethical online behavior.<br>• Females were more likely to post photographs of students at social events.	Observing potential instances of inappropriate social media behavior among certain dental students highlights the need for additional education; thus, it is essential to provide training to undergraduate dental students focusing on the proper use of social media and the effective management of online risks.
Kim & Choi (2021) /South Korea [5]	To investigate the frequency of cyberbullying among nursing students in clinical practice and assess its influence on their understanding of	Junior and senior nursing students with clinical experience at two universities<br><br>n = 291	Questionnaire	• Most students did not receive prior education on bullying within the university curriculum and none received cyberethics education.<br>• Nurses and classmates were the most common cyberbullies.<br>• Cyberethics awareness showed	Clinical workplaces and nursing universities should work together to address cyberethics through a multilateral cooperation system and provide effective practical nursing education to nurses and students to reduce cyberbullying.

	cyberethics			significant negative correlations with cyberbullying related to clinical practices and cyber-anonymity but significant positive correlations with the perceived seriousness of cyberbullying.	
Knott & Wassif (2018) /UK [63]	To explore the perspectives of first year Bachelor of Dental Surgery (BDS) students and their use of social media platforms, while gathering their viewpoints on professional behaviors in online contexts	Bachelor of dentistry program<br><br>Phase 1: (questionnaires) n = 22 first year BDS students who were five months into their BDS program<br><br>Phase 2: (semi-structured interviews) n = 4 BDS students from phase 1	Questionnaire and semi-structured interviews	• All participants used social media, primarily WhatsApp, Facebook, and Snapchat.<br>• Despite some participants showing superficial knowledge of what is and is not professional to post via social media, most students were not fully aware of the legal and ethical implications of social media use.	Educators are confronted with the dual challenge and opportunity of integrating information on professionalism and ethics within the context of social media use, while the creation of pertinent real-life content for cyberethics education presents challenges; however, schools should actively collaborate to share knowledge and enrich students' learning prospects.
Lee et al. (2021) /South Korea [30]	To explore unprofessional behaviors in YouTube videos posted by medical students	n = 79 YouTube videos uploaded by Korean medical students between March 14th and April 25th, 2020	Quantitative and qualitative analysis of video characteristics and content	• The most common type of content was video blogs, primarily focusing on personal and daily student life in medical schools or teaching hospitals.<br>• Approximately 20% of the collected videos contained at least one undesirable behavior: (1) Failure to engage in academic tasks (2) Disrespectful behaviors (3) Poor self-awareness.<br>• Although one highly concerning behavior of sexist slurs was classified under 'disrespectful behaviors,' the majority of the coded behaviors were of lesser severity.	The accessibility of unprofessional content posted online by medical students poses a potential risk for adversely affecting the entire medical profession; thus, medical education should include guidance on ethical public video uploads that uphold medical professionalism.

Lie et al. (2013) /USA [40]	To examine attitudes, self-reported behaviors, and intended actions related to medical students' use of online social media following an educational intervention	Medical students entering their first year<br><br>n = 180<br><br>Post session evaluation: n = 180<br>Four-month follow-up survey: n = 115	Written student reflections, course evaluations, and a four-month follow-up survey	• Medical students have a strong online presence, and they are open to discussing this topic and are also ready to edit and monitor their online presence during training.<br>• Participation in the intervention resulted in reflection, increased professional role awareness, intention to change future online activities and monitor the activity of other medical students, which remained present at the follow-up evaluation.<br>• Three domains emerged: (1) Immediate action (2) Intended future action, (3) Value change.	A theoretical framework is proposed to guide future cyberethics education, integrating discussions on social media use into existing professionalism or medical courses through a learner-centered approach and supportive learning environments; this intervention and framework could enhance faculty understanding of students and foster stronger mentoring connections in the future.
Mosalanejad et al. (2014) /Iran [52]	To investigate and describe the experiences of the students enrolled in a University of Medical Sciences in relation to cyber-systems ethics	Students in medicine, nursing, operating room, and anesthesiology<br><br>n = 25	In-depth, semi-structured focus groups	• Two main themes emerged: (1) Ethical implications in virtual systems, (2) Ethics development factors.<br>• Moral cyber experiences were influenced by factors such as the students' personal values, cultural background, and the institutional context.	The development of ethics training that addresses the optimal utilization of cyberspace and technology across diverse scientific domains holds significance; concurrently, families, schools, and universities should collaboratively take initiatives to cultivate ethical practices among internet users, with a particular focus on students.
Nieminen et al (2022) /Malaysia and Finland [53]	To and compare the perceptions of Malaysian and Finnish dental students on e-professionalism and explore challenges faced using communication technology	Dental students enrolled in 5-year undergraduate dentistry programs in Malaysia and Finland<br><br>n = 613	English and Finnish Questionnaire	• Malaysian and Finnish students displayed different perceptions and behaviors regarding social media activities, with Malaysian students identifying violations in patient confidentiality as more serious.<br>• Finnish students posted more objectionable or unsuitable content than Malaysian students.<br>• Students from both countries reported that they had never posted inappropriate content, but encountered inappropriate content posted by their	Schools must integrate comprehensive digital professionalism awareness training into their curricula, encompassing diverse e-professionalism themes; this training should enable students to grasp the boundaries of professional conduct on social media, distinguish between personal and professional online personas, and be adaptable to various cultural contexts.

				peers.<br>• Both groups had a positive outlook on the use of social media, but Malaysian students placed emphasis on the benefits and the importance of responsible usage compared to Finnish students.	
Nyangeni et al. (2015) /South Africa [71]	To explore nursing students' perceptions of responsible social media usage, while identifying the various ways in which they employ social media within the clinical environment	Bachelor of Nursing students including native and international students<br><br>n = 12	Semi-structured interviews	Two main themes emerged: (1) There is no awareness of responsible use of social media amongst nursing students, (2) There are blurred boundaries between private and public roles and a lack of accountability.	Comprehensive real-world cyberethics education program may be necessary to help students understand cyberethics, digital professionalism, patient privacy and confidentiality, potential negative impacts of their online activities, and the boundaries between personal and professional life.
O'Connor et al. (2022) /UK [56]	To understand the views of undergraduate nursing students towards digital professionalism on social media	Bachelor of Nursing students at one university<br><br>n = 112	Mixed method questionnaire with multiple choice questions and open-ended responses	• Nursing students had some understanding of digital professionalism and the potential risks associated with their social media activities.<br>• Confusion existed due to blurred boundaries between personal and professional lives in cyberspace and challenges in maintaining digital professionalism on social media, such as balancing personal and professional identities and dealing with negative comments or feedback.	Education of nursing students should include (1) Professional values and behaviors on social media, (2) Proper communication, interaction, and information sharing skills on various social media platforms; nursing facilities should provide clear policies and guidelines on social media to address cybercivility and prevent legal and ethical violations online.
O'Connor et al. (2021) /Not applicable [27]	To identify and synthesize literature on educating health care students and practitioners about digital professionalism on social media	11 included studies published between 2010 - 2018, across three countries including USA, UK, and Canada	Analysis guide by Braun and Clarke's six phases of thematic analysis	• Across included studies, digital professionalism was taught to nursing, medical and allied health professions students using face-to face, hybrid, and online approaches.<br>• The creation and delivery of digital professionalism education was impacted by the amount of time faculty	Implementing education on digital professionalism presents various barriers (e.g., timeliness, generational gaps, poor digital literacy) and facilitators (e.g., well-defined boundaries, real-world example, and clear guidelines); course development should include

				were able to dedicate to the development and organization of curricula, delivery of teaching, and providing student and clinician support.<br>• Two out of 11 included studies were guided by a pedagogical theory.	promoting digital literacy knowledge and skills, incorporating professional policies relevant to health care disciplines, using real-world case studies, engaging students and clinicians in curricular development, and providing clear descriptions of faculty availability and interactivity on social media.
Oakley & Spallek (2012) /USA [61]	To describe the impact of social media in current health care settings, emphasizing the potential advantages and obstacles associate with its utilization within these settings	N/A	N/A	• Potential benefits of social media in dental education include enhancing communication, providing patient-centered information, and delivering health promotion content .<br>• Potential challenges involve posting unprofessional or patient specific content, lack of knowledge of privacy settings, and limited guidance on proper regulations or educational policies.	Schools need to encompass discussions about the appropriateness of posting online content, coupled with the provision of professional guidelines for students' online activities, given the limited discourse in dental education literature concerning the implications of social media.
Peck (2014) /USA [62]	To explore the legal and ethical ramifications of incorporating social media as an educational tool in nursing education, while discerning optimal practices and guidelines for the conscientious utilization of social media in this context	N/A	N/A	• Social media may enhance nursing education by promoting collaboration, engagement, and professional development.<br>• Integration of social media requires an approach that emphasizes ethical and professional use.<br>• Evaluation and assessment of social media integration can help identify areas for improvement and promote meaningful use.	Incorporating social media into the curriculum, nurse educators should consult the NCSBN White Paper: A Nurse's Guide to the Use of Social Media and refer students to the ANA's standards of nursing practice; faculty should provide instruction on ethical social media use, assess its impact on lectures and student learning, and adapt teaching approaches accordingly, emphasizing that faculty training and support are pivotal for the successful integration of social media in nursing education.
Spallek et al. (2015) /USA [41]	To describe curricular constructs surrounding teaching and learning	N/A	N/A	• There are risks associated with social media use in dental students (e.g., misinterpretations, permanence,	Dental schools should integrate social media education into their curricula to prepare students for the

	about social media in dental education			disinhibited self-disclosure, negative feedback, and libel).<br>• There are various ways that social media can enhance dental education, such as promoting student engagement and collaboration, providing reflection opportunities, and facilitating communication.	realities of modern professional practice, with a specific emphasis on ethical considerations related to the advent of social media; this educational approach should encompass discourse on cyberethics, regulatory frameworks, and the exemplification of professional roles.
Viskic et al. (2021) /Croatia [54]	To escribe and compare social media use habits, attitudes, and opinions of medical and dental students toward e-professionalism	Medical and dental students<br><br>n = 698; n = 411 medical students; n = 287 dental students	Questionnaire	• Social media use is universal and both groups had a high awareness of e-professionalism.<br>• Unprofessional behavior in both student groups was low.<br>• Dental students were more desensitized to patient images and more likely to interact with patients online.	Guidelines should be established to facilitate the integration of e-professionalism into medical and dental curricula, promoting consistent ethical and legal foundations for practice among both medical and dental students.
Westrick (2016) /USA [55]	To discuss the promotion of professionalism in nursing students while using social media	N/A	N/A	• Based on the two legal cases presented involving students who were dismissed from nursing program due to social media misuse, it is recommended that schools have explicit policies regarding the use and misuse of electronic communications and social media and delineating the consequences for policy violations.<br>• Promoting e-professionalism among nursing students through education and training is an important strategy for preventing negative effects of social media.	Nurse educators and administrators should develop specific polices and guidelines related to social media use, while considering the potential effectiveness of simulations and case studies as educational tools to exemplify desired behaviors in a safe environment.
Won (2022) /South Korea [50]	To describe cyberincivility among nursing students, particularly in remote learning environments and explore their related	National sample of nursing enrolled in universities who had taken at least one remote	The cyber non-citizenship survey tool and focus group interviews	Nursing students encountered various forms of uncivil behavior in online spaces, leading to the identification of three themes: (1) Inappropriate learning attitudes during remote learning, (2) Lack of consideration for others, and (3)	Designing educational programs should be geared towards diminishing the normalization of uncivil behaviors in online spaces among nursing students, concurrently highlighting the

	perspectives	learning course since March 2020<br><br>n = 142		Limited interest in privacy policies.	benefits of learning cybercivility to reshape their perceptions.
Yunker (2021) /USA [49]	To announce the introduction of an EthicsPoint tool used to report inappropriate behavior during Physician Assistant Education Association (PAEA) assessment exams	N/A	N/A	The reporting tool aims to promote responsible behavior and ethical conduct when physician assistant (PA) students are testing online by encouraging individuals to partake in ethical test-taking and report instances of not following ethical principles.	By adhering to these guidelines, PA students can demonstrate proper cyberethics and support a culture of honest, responsible, and ethical behavior in virtual testing environments.
Zhu et al. (2021) /China [58]	To investigate nursing students' usage, professionalism, and attitudes toward social media	Undergraduate nursing students at one Chinese University<br><br>n = 654	Questionnaire	• More than half of participants reported the importance of schools developing a social media and professionalism course for nursing students.<br>• Most participants reported being unaware of professional nursing standards of behavior online for health care workers.<br>• Nursing students used social media mainly for academic-related purposes and had positive attitudes towards it.	Initiating ethics education that centers around social media should commence during the first year of nursing school, concurrently with the establishment of policies and guidelines by nursing schools for the utilization of social media by their students.
