# Supplementary material for: Exploration of Cyberethics in Health Professions Education: A Scoping Review
Source: Int J Environ Res Public Health. 2023 Nov 10;20(22):7048. doi: 10.3390/ijerph20227048 (PMC10671151; doi:10.3390/ijerph20227048)
Supplement: Supplementary file 1 [file ijerph-20-07048-s001.zip › Table S3_Conceptualization of cyberethics of the included documents.pdf]

Table S3. Conceptualization of Cyberethics Identified in the Included Documents (*n* = 37).

Author(s) (Year)	Related Cyberethics Concept or Term/Definition	Application to Conceptual or Theoretical Frameworks/Models
American Association of Colleges of Pharmacy (AACP) (2022) [34]	Not reported; reference to cyberbullying and spreading of misinformation as misuse of social media	Not specified but resource guide is consistent with the principles of professionalism in healthcare and ethical principles of autonomy, beneficence, non-maleficence, and justice
Aplin-Snider et al. (2021) [46]	Not reported; exploration of academic integrity and dishonesty (e.g., willingness to report cheating)	Not specified but methodology and research questions suggest that it was informed by a constructivist paradigm
Azulay Chertok et al. (2014) [47]	Not reported; focus on academic integrity and plagiarism in an online setting	Not reported
Barlow et al. (2015) [35]	*Unprofessional content*: an accessible online depiction of illegal activity, overt intoxication or illicit drug use, or the posting of patient information	Not reported
Burlington (2020) [48]	*Academic integrity*: the act of being honest and completing work without intentionally plagiarizing	Social Cognitive Theory; Theory of Student Cheating and Plagiarism
Cha (2017) [51]	*Information ethics*: guidelines to address various ethical challenges that arise in the information society, from the creation to removal of information on various networks	Theory of Planned Behavior
Chester et al. (2017) [42]	*Patient targeting googling*: when a health care professional uses a social media site or search engine to access patient information online	Not specified but authors suggest health care professionals may use a pragmatic framework, such as the one proposed by Clinton et al. (2010)
Clark et al. (2012) [31]	*Cyberbullying and incivility*: uncivil behaviors in the online learning environment (e.g., trolling, flaming)	Not reported
Commission on Collegiate Nursing Education (CCNE) (2021) [43]	Not reported; focus on the impact of technology in health care and how nurse residents should be prepared to care for patients in a virtual environment	Not reported
de Peralta et al. (2019) [60]	Not reported; discussion of the positive and negative impact of social media on dental professionalism	Reference to the American Medical Association (AMA) principles of medical ethics and social media use
Duke et al. (2017) [32]	*E-professionalism*: the internal and external attitudes and behaviors which reflect traditional professionalism paradigms presented via digital media	Not reported
Eichler (2018) [36]	Not reported; focus on the presentation of dental students professionally while using social media	Not specified but the blog post is consistent with the principles of professionalism in healthcare and ethical principles of autonomy, beneficence, non-maleficence, and justice
Ellis (2016) [28]	*Academic dishonesty*: purposely attempting to falsify, fabricate or tamper with data, information, records, or any other material that is relevant to participation in any course, laboratory, or other academic exercise or function<br>*Academic integrity*: undertaking scholarly activity in an open,	Systems Theory; Espoused Theory; Model I and Model II Theory; and Change Theory

	honest, and responsible manner	
Englund et al. (2012) [44]	Not reported; focus on unprofessional social media posts (e.g., excessive drinking, affiliation with unacceptable social groups)	Authors used the American Nurses Association (ANA) Code of Ethics (2001) to guide study and develop survey tool
Gormley et al. (2021) [37]	*Digital professionalism*: no definition reported	Theory of Planned Behavior used to interpret focus group transcripts; British Psychological Society guidance used to analyze inappropriate behavior; Qualified framework analysis used to generate themes
Henry & Molnar (2013) [29]	Not reported; focus on unprofessional online content (e.g., substance abuse, sexism, racism)	Not reported
Kamarudin et al. (2022) [38]	*E-professionalism*: behaviors and attitudes representing professionalism which are manifested through an online environment	Not reported
Karveleas et al. (2021) [45]	*E-professionalism*: behaviors and attitudes reflecting typical professionalism examples that are represented through social media	Not specified but authors report connection between e-professionalism and principles such as altruism, integrity, compassion, respect, ethics, and accountability
Keating (2016) [39]	Not reported; focus on the presentation of medical students in an ethical manner when using social media platforms	Not specified but guidelines are consistent with the ethical principles of autonomy, beneficence, non-maleficence, justice, and respect for diversity
Kenny & Johnson (2016) [33]	Not reported	Not specified but authors reference the United Kingdom General Dental Council (GDC) guidance on social media for guidance in professional online behavior
Kim & Choi (2021) [5]	*Cyberethics*: a standardized system used to delineate the morality of behavior in cyberspace with a focus on safeguarding intellectual freedom, expression, and privacy	Author created framework depicting the internal and external predictors of cyber ethics awareness among nursing students
Knott & Wassif (2018) [63]	Not reported	Not specified but authors describe the GDC guidance on social media as it pertains to professionalism and fitness to practice
Lee et al. (2021) [30]	*Digital professionalism/ e-professionalism*: actions and attitudes which reflect traditional professionalism paradigms that one manifests through digital media	Not reported
Lie et al. (2013) [40]	Not reported; focus on the unprofessional conduct of physicians and medical students online	The study intervention was framed using the AMA guidelines (2011) for professionalism in the use of social media<br>Authors formulated a theoretical framework based on their findings to guide educators in integrating cyberethics pedagogy into established curricula
Mosalanejad et al. (2014) [52]	*Cyberethics*: the philosophical study of ethical behavior within interdisciplinary computer networks and its impact on individuals and society	Not reported

Nieminen et al. (2022) [53]	*E-professionalism*: attitudes, identity, and behaviors represented through digital media that reflect professionalism paradigms	Not reported
Nyangeni et al. (2015) [71]	*Unethical use of social media*: use of social media that can damage personal integrity, privacy and online security, nurse-patient relationships, nurse-colleague relationships, and opportunities for employment	Not specified but authors mention that the ethical principle of confidentiality is an ethical and legal responsibility of nurses to uphold
O'Connor et al. (2022) [56]	*Digital professionalism*: private and outward attitudes and behaviors that reflect traditional professionalism paradigms which are displayed through online media	Authors reference the Nursing and Midwifery Code (NMC) which outlines professional guidelines required for nurses to be accountable and practice safely with respect and dignity
O'Connor et al. (2021) [27]	*Digital professionalism*: private and outward attitudes and behaviors that reflect traditional professionalism paradigms which are displayed through online media	Out of 11 include studies, only two were underpinned by a pedagogical theory
Oakley & Spallek (2012) [61]	Not reported; focus on challenges of using social media including unprofessional online activity and promoting ethical online interactions	Authors describe the importance of using the AMA guidelines (2011) for professionalism in the use of social media to maintain patient confidentiality and patient/provider boundaries
Peck (2014) [62]	Not reported	Not specified but author encourages use of ANA social networking principles toolkit and the NCSBN White Paper: "A Nurse's Guide to the Use of Social Media"
Spallek et al. (2015) [41]	Not reported	Not specified but authors highlight the PRIP acronym (Privacy, Respect, Intent, Perception) and the American Dental Association (ADA) Principles of Ethics and Code of Professional Conduct for online professionalism guidance
Viskic et al. (2021) [54]	*E-professionalism*: views and actions that are representative of traditional professionalism models through digital media	Not reported
Westrick (2016) [55]	*E-professionalism*: ethical and professional use of electronic and social media platforms by nursing students	Authors refer to the ANA Code of Ethics for Nurses (2015) for guidance on online ethics and NCSBN, HIPAA, and the HITECH Act to protect patient confidentiality in virtual settings
Won (2022) [50]	*Cyberincivility*: behaviors that violate ethical norms and standards in cyberspace (e.g., cyberbullying, cyber-harassment)	Not reported
Yunker (2021) [49]	*Inappropriate testing behavior*: unethical collection or dissemination of online exam information	Not specified but tool is consistent with the principles of professionalism in health care and ethical principles of autonomy, beneficence, non-maleficence, and justice
Zhu et al. (2021) [58]	*Professionalism on social media*: attitudes and behaviors reflecting conventional professionalism theories which become apparent through social media	Authors reference the NMC to guide professionalism in digital communication
