# Supplementary figures and images for: Exploration of Cyberethics in Health Professions Education: A Scoping Review
Source: Int J Environ Res Public Health. 2023 Nov 10;20(22):7048. doi: 10.3390/ijerph20227048 (PMC10671151; doi:10.3390/ijerph20227048)

**Figure S3.** Conceptualization of cyberethics in literature.

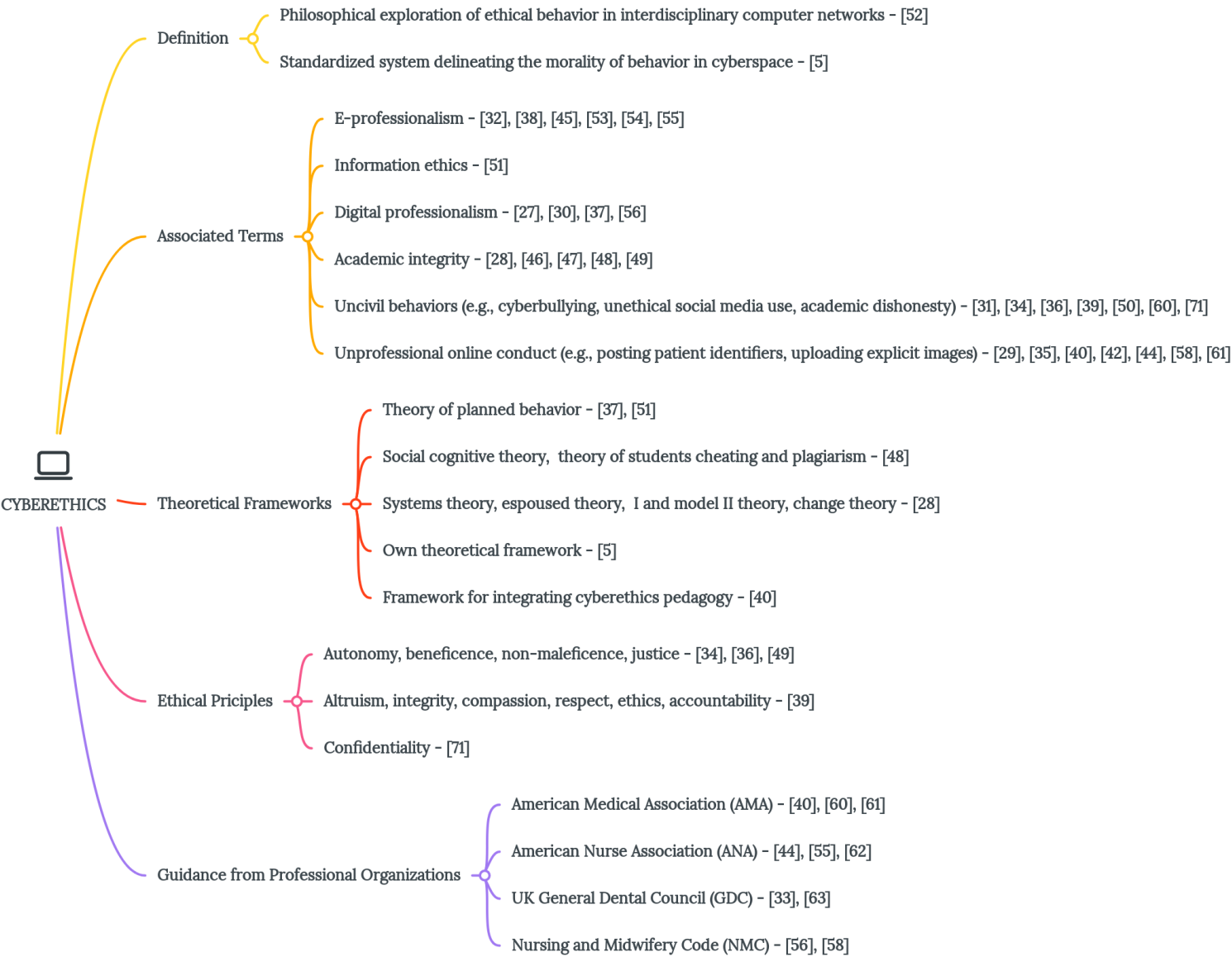

Supplement: Supplementary file 1 [file ijerph-20-07048-s001.zip › Figure S3_Conceptualization of cyberethics in literature.pdf]
